# Human Pluripotent Stem Cell-Derived Astrocyte Functionality Compares Favorably with Primary Rat Astrocytes

**DOI:** 10.1523/ENEURO.0148-24.2024

**Published:** 2024-09-12

**Authors:** Bas Lendemeijer, Maurits Unkel, Hilde Smeenk, Britt Mossink, Sara Hijazi, Sara Gordillo-Sampedro, Guy Shpak, Denise E. Slump, Mirjam C.G.N. van den Hout, Wilfred F.J. van IJcken, Eric M.J. Bindels, Witte J.G. Hoogendijk, Nael Nadif Kasri, Femke M.S. de Vrij, Steven A. Kushner

**Affiliations:** ^1^Department of Psychiatry, Erasmus University Medical Center, Rotterdam 3015 AA, The Netherlands; ^2^Department of Psychiatry, Columbia University, New York, New York 10027; ^3^Stavros Niarchos Foundation (SNF) Center for Precision Psychiatry & Mental Health, Columbia University, New York, New York 10027; ^4^Department of Human Genetics, Radboud University Medical Center, Nijmegen 6525GA, The Netherlands; ^5^Department of Cell Biology, Center for Biomics, Erasmus University Medical Center, Rotterdam 3015AA, The Netherlands; ^6^Department of Hematology, Erasmus University Medical Center, Rotterdam 3015AA, The Netherlands; ^7^ENCORE Expertise Center for Neurodevelopmental Disorders, Erasmus University Medical Center, Rotterdam 3015AA, The Netherlands

**Keywords:** astrocyte, coculture, developmental biology, electrophysiology, in vitro, iPSC

## Abstract

Astrocytes are essential for the formation and maintenance of neural networks. However, a major technical challenge for investigating astrocyte function and disease-related pathophysiology has been the limited ability to obtain functional human astrocytes. Despite recent advances in human pluripotent stem cell (hPSC) techniques, primary rodent astrocytes remain the gold standard in coculture with human neurons. We demonstrate that a combination of leukemia inhibitory factor (LIF) and bone morphogenetic protein-4 (BMP4) directs hPSC-derived neural precursor cells to a highly pure population of astroglia in 28 d. Using single-cell RNA sequencing, we confirm the astroglial identity of these cells and highlight profound transcriptional adaptations in cocultured hPSC-derived astrocytes and neurons, consistent with their further maturation. In coculture with human neurons, multielectrode array recordings revealed robust network activity of human neurons in a coculture with hPSC-derived or rat astrocytes [3.63 ± 0.44 min^−1^ (hPSC-derived), 2.86 ± 0.64 min^−1^ (rat); *p *= 0.19]. In comparison, we found increased spike frequency within network bursts of human neurons cocultured with hPSC-derived astrocytes [56.31 ± 8.56 Hz (hPSC-derived), 24.77 ± 4.04 Hz (rat); *p *< 0.01], and whole-cell patch-clamp recordings revealed an increase of postsynaptic currents [2.76 ± 0.39 Hz (hPSC-derived), 1.07 ± 0.14 Hz (rat); *p *< 0.001], consistent with a corresponding increase in synapse density [14.90 ± 1.27/100 μm^2^ (hPSC-derived), 8.39 ± 0.63/100 μm^2^ (rat); *p *< 0.001]. Taken together, we show that hPSC-derived astrocytes compare favorably with rat astrocytes in supporting human neural network activity and maturation, providing a fully human platform for investigating astrocyte function and neuronal-glial interactions.

## Significance Statement

Astrocytes are essential for the formation and integrity of neuronal microcircuits. Due to species differences within the astrocyte lineage, there has been considerable effort invested in developing methods to establish hPSC-derived astrocytes in vitro. However, in a coculture system with hPSC-derived neurons, supplementation with primary rodent astrocytes remains the gold standard, thereby limiting the potential benefits of an entirely human cellular system. This work benchmarks the functionality of cocultures of hPSC-derived neurons supplemented with either primary rat astrocytes or hPSC-derived astrocytes. We found that hPSC-derived astrocytes compare favorably with primary rat astrocytes, providing the opportunity to establish a fully human system suitable for investigating human neurodevelopment and neuropsychiatric disease modeling.

## Introduction

Astrocytes are required for microcircuit function and no longer considered to merely provide structural support for neurons (Allen and Barres, 2009). Astrocytes provide neurons with a critical source of metabolites (Bélanger et al., 2011), regulate blood flow (Macvicar and Newman, 2015), maintain the blood–brain barrier (Abbott et al., 2006), regulate inflammation in the central nervous system (Giovannoni and Quintana, 2020), facilitate synapse formation (Allen and Eroglu, 2017), modulate neuronal network activity (Deemyad et al., 2018), and contribute to memory encoding (Kol et al., 2020; Sun et al., 2024). Astrocyte morphological complexity is one of the distinguishing features between the human and rodent brain (Oberheim et al., 2006). Moreover, increasing evidence has highlighted robust functional differences between rodent and human astrocytes. Compared with their rodent counterparts, human astrocytes exhibit distinct calcium responses (Zhang et al., 2016) and enhanced synaptogenesis (Diniz et al., 2012).

Guided differentiation of human pluripotent stem cells (hPSCs) provides the opportunity to study the development of the human brain in vitro (Astick and Vanderhaeghen, 2018). Multiple protocols have been reported for differentiation of human stem cells into neurons (Marchetto et al., 2010; Miller et al., 2013; Gunhanlar et al., 2018). A widely adopted method that yields a pure culture of excitatory neurons through forced *Ngn2*-overexpression requires coculture with astrocytes to ensure neuronal survival and maturation (Zhang et al., 2013). The currently available sources of astrocytes for coculture with hPSC-derived neurons include primary rodent astrocytes (the current gold standard in the field; Hulme et al., 2022; S. Wang et al., 2022; Bullmann et al., 2024) or human pluripotent stem cell-derived astrocytes (Krencik et al., 2017; Jovanovic et al., 2023). Using rodent astrocytes can be problematic for many experimental designs, due to the genomic and functional differences between human and rodent cells. Two recent studies (Garcia et al., 2019; Hedegaard et al., 2020) have demonstrated that supplementing additional iPSC-derived astrocytes to a neural culture containing neurons, astrocytes, and NPCs improves the electrophysiological properties of neurons. Other studies describe increased synaptogenesis or enhanced neuronal electrophysiological maturation; however, this is often compared with a neuronal culture without astrocytes (Krencik et al., 2017; Jovanovic et al., 2023) or they were unable to fully recapitulate neuronal electrophysiological properties in a coculture with hPSC-derived astrocytes compared with primary murine astrocytes (Shih et al., 2021). Generally speaking, readouts to validate hPSC-derived astrocyte protocols are focused on astrocyte characterization by comparing their transcriptomic profile to primary human or rodent astrocytes (Caiazzo et al., 2015; Kondo et al., 2016; Sloan et al., 2017; Tcw et al., 2017; di Domenico et al., 2019).

Here, we present a functional comparison of human and rodent astrocytes in coculture with human neurons using a modified protocol to differentiate hPSC-derived neural progenitor cells (NPCs) into functional cortical astrocytes. We have systematically compared the electrophysiological properties of hPSC-derived neurons in coculture with primary rat astrocytes or hPSC-derived astrocytes and show that hPSC-derived astrocytes are able to support a higher level of neuronal activity compared with primary rat astrocytes. Using immunocytochemistry, (single-cell) RNA sequencing, and flow cytometry, we demonstrate that hPSC-derived astroglia express the canonical astrocytic markers and at similar levels when compared with primary rat astrocytes. Morphological analysis shows that hPSC-derived astroglia display hominid morphological features. Both in a mono- and in a coculture with human neurons, hPSC-derived astrocytes are larger compared with rat astrocytes. Following xenotransplantation and integration into the local microenvironment of a mouse brain, hPSC-derived astrocytes maintain this hominid morphological characteristic and are larger when compared with resident mouse astrocytes. We show that synapse development and spontaneous excitatory postsynaptic currents are increased in cocultures of human neurons and hPSC-derived astrocytes compared with cocultures of human neurons with rat astrocytes. Taken together, our data demonstrate that hPSC-derived astrocytes promote neuronal maturation and synaptic function, thus eliminating the need for their rodent counterparts in human neural coculture systems.

## Materials and Methods

### Resource availability

Further information and requests for resources and reagents should be directed to the corresponding authors, Steven A. Kushner (sk2602@cumc.columbia.edu) or Femke M.S. de Vrij (f.devrij@erasmusmc.nl).

### Experimental model and subject details

All cells were maintained in an incubator at 37°C/5% CO_2_. Human PSCs were expanded in hES medium (Table 1) on a feeder layer of mouse embryonic fibroblasts. Four independent hPSC lines were used, three induced pluripotent stem cell lines [WTC-11 provided by Bruce R. Conklin (The Gladstone Institute and UCSF; Miyaoka et al., 2014), RRID:CVCL_Y803 (iPS1), two in-house previously established control lines (de Vrij et al., 2019; Erasmus MC iPS Core Facility EMC13i955-3, male, age 57, iPS2; EMC14i96-1, female, age 54, iPS3)] and an embryonic stem cell (ES) line [SA001 (Adewumi et al., 2007), RRID:CVCL_B347, male]. Primary rat astrocytes (ScienCell, SCCR1800) and iCell GlutaNeurons (Cellular Dynamics, R1034) were obtained from the manufacturer and maintained according to instructions.

**Table 1.** Overview of media and reagents used

Name	Reagents	Manufacturer, catalog #
hES Medium	Advanced DMEM/F12	Thermo Fisher Scientific, 12634010
	20% KnockOut Serum Replacement	Thermo Fisher Scientific, 10828028
	1% MEM-NEAA	Thermo Fisher Scientific, 11140035
	7 nl/ml β-mercaptoethanol	Sigma-Aldrich, M7522
	1% ʟ-Glutamine	Thermo Fisher Scientific, 25030024
	1% Penicillin-Streptomycin	Thermo Fisher Scientific, 15140122
	10 ng/ml basic Fibroblast Growth Factor	Merck, GF003AF
Neural Induction Medium	Advanced DMEM/F12	Thermo Fisher Scientific, 1634010
	1% N-2 Supplement	Thermo Fisher Scientific, 17502048
	2 μg/ml Heparin	Sigma-Aldrich, H3149
	1% Penicillin-Streptomycin	Thermo Fisher Scientific, 15140122
NPC Medium	Advanced DMEM/F12	Thermo Fisher Scientific, 12634010
	1% N-2 Supplement	Thermo Fisher Scientific, 17502048
	2% B-27 minus RA Supplement	Thermo Fisher Scientific, 12587010
	1 μg/ml Laminin	Sigma-Aldrich, L2020
	1% Penicillin-Streptomycin	Thermo Fisher Scientific, 15140122
	20 ng/ml basic Fibroblast Growth Factor	Merck, GF003AF
Astrocyte Medium	Advanced DMEM/F12	Thermo Fisher Scientific, 12634010
	1% N-2 Supplement	Thermo Fisher Scientific, 17502048
	2% B-27 minus RA Supplement	Thermo Fisher Scientific, 12587010
	1 μg/ml Laminin	Sigma-Aldrich, L2020
	1% Penicillin-Streptomycin	Thermo Fisher Scientific, 15140122
	10 ng/ml Bone morphogenic protein 4	BioVision, 4578
	10 ng/ml Leukemia Inhibitory Factor	PeproTech, 300-05
iCell Medium	BrainPhys Neuronal Medium	StemCell Technologies, 05790
	1% N-2 Supplement	Thermo Fisher Scientific, 17502048
	1% iCell Nervous System Supplement	Cellular Dynamics, M1031
	2% iCell Neural Supplement B	Cellular Dynamics, M1029
	1 μg/ml Laminin	Sigma-Aldrich, L2020
	1% Penicillin-Streptomycin	Thermo Fisher Scientific, 15140122
*Ngn2 *Day 1 Medium	Advanced DMEM/F12	Thermo Fisher Scientific, 1634010
	1% N-2 Supplement	Thermo Fisher Scientific, 17502048
	1% MEM-NEAA	Thermo Fisher Scientific, 11140050
	1% Penicillin-Streptomycin	Thermo Fisher Scientific, 15140122
	4 µg/ml Doxycycline	Sigma-Aldrich, D5207
	10 ng/ml NT3	PeproTech, 450-03
	10 ng/ml BDNF	ProSpec, CYT 207
	0.2 µg/ml Laminin	Sigma-Aldrich, L2020
*Ngn2* Medium	Neurobasal Medium	Thermo Fisher Scientific, 21103049
	2% B-27 minus RA Supplement	Thermo Fisher Scientific, 12587012
	1% GlutaMAX	Thermo Fisher Scientific, 35050061
	4 µg/ml Doxycycline	Sigma-Aldrich, D5207
	10 ng/ml NT3	PeproTech, 450-03
	10 ng/ml BDNF	ProSpec, CYT 207
	0.1 μg/ml Primocin	InvivoGen, ant-pm-1

All mouse experiments were approved by the local animal welfare committee, and mice were kept under standard housing conditions with *ad libitum* access to food and water. Both male and female immunodeficient *Rag2*^−/−^ BALB/c (Jackson, 014593) mice were used for transplantation studies and killed between 4 and 40 weeks of age.

All procedures with human tissue were performed with the approval of the Medical Ethical Committee of the Erasmus MC Rotterdam, including written informed consent of all subjects for brain donation in accordance with Dutch license procedures and the Declaration of Helsinki. Fresh-frozen postmortem tissue blocks containing the middle frontal gyrus (BA9) from three donors [61 (female), 79 (male), and 81 (female) years old] were obtained from the Erasmus MC Department of Pathology.

### Experimental design and statistical analysis

For each experiment, human astroglia were derived from cryopreserved NPCs that had previously been established from four different hPSC lines and specific lines, and total *n* is indicated in the figure legends. Cultures containing hPSC-derived or rat astrocytes were grown in parallel to control for batch effects. Bulk RNA sequencing data was analyzed using Fisher's exact test and FDR corrected for multiple testing. Single-cell RNA sequencing data was analyzed using Seurat's implementation of DESeq2. The parameters logfc.threshold and min.pct were set to 0. *p* values adjusted for multiple testing error were used for thresholding significance at *p *< 0.05. For functional studies, statistical comparisons were performed using Fisher's exact test, two-tailed *t* test, or analysis of variance (ANOVA), as indicated. Data are expressed as mean ± SEM, unless otherwise specified. The threshold for significance was set at *p *< 0.05 for all statistical comparisons.

### Astroglia differentiation

**Figure 1.**
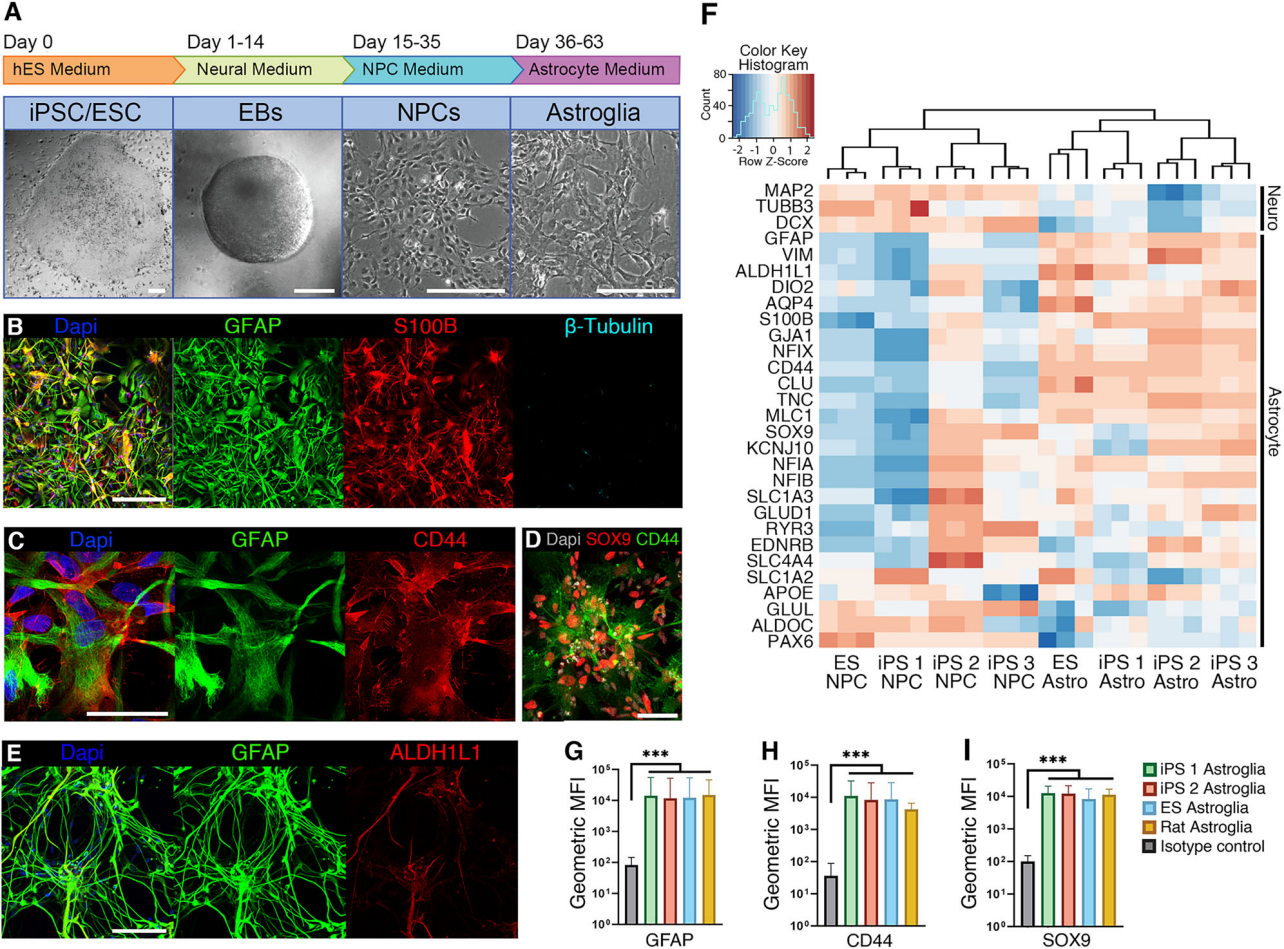
Astrocyte differentiation leads to a pure population of human astroglia in four weeks. ***A***, Schematic representation of the differentiation protocol with representative differential interference contrast images of the different stages (scale bars, 200 μm). ***B***, Staining for astrocytic markers 4 weeks after NPC stage (GFAP, green, and S100β, red) and an early neuronal marker (β-tubulin, cyan; scale bar, 200 μm). Immunofluorescent labeling of astrocytic markers of all cell lines used in this study is displayed in Extended Data Figure 1-1. Differentiation efficiency of astrocyte medium containing BMP4 and LIF, only BMP4, only LIF, or no additional growth factors is quantified in Extended Data Figure 1-2. ***C***, Staining for glial membrane marker CD44 (red) and GFAP (green; scale bar, 50 μm). ***D***, Nuclear astrocytic marker SOX9 is shown in red, together with CD44 in green (scale bar, 50 μm). ***E***, A subset of astroglia (GFAP, green) stains positive for ALDH1L1 (red) a mature astrocyte marker (scale bar, 100 μm). ***F***, Clustering of RNA sequencing data of three differentiation batches of NPCs to astroglia from four different human pluripotent stem cell lines (iPS1, iPS2, iPS3, and ES). Genes of interest are depicted on the left, on the top 3 neuronal genes and below 26 astrocyte genes. See Extended Data Figure 1-3 for a Principal Component Analysis plot of bulk RNA sequencing results. ***G–I***, Flow cytometry data from iPS1, iPS2, and ES lines shows that 5-week-old astroglia cultures show comparable geometric mean fluorescence intensity (MFI) to primary rat astrocytes for GFAP (***G***), CD44 (***H***), and SOX9 (***I***). Fluorescence intensity histogram plots of these data are depicted in Extended Data Figure 1-4.

We validated our protocol using four independent human pluripotent stem cell (hPSC) lines: induced pluripotent stem cell lines [WTC-11 provided by Bruce R. Conklin (The Gladstone Institute and UCSF ;Miyaoka et al., 2014), RRID:CVCL_Y803 (iPS1), two in-house previously established control lines (de Vrij et al., 2019; Erasmus MC iPS Core Facility EMC13i955-3, male, age 57, iPS2; EMC14i96-6, female, age 54, iPS3)] and an embryonic stem cell (ES) line [SA001 (Adewumi et al., 2007), RRID:CVCL_B347, male]. All hPSC lines and their derivatives underwent regular microarray-based screening for structural genomic variation and were screened for mycoplasma every other month. Pluripotent stem cells were differentiated to NPCs in 40 d and cryopreserved as previously described (Gunhanlar et al., 2018) with slight modifications (Extended Data Fig. 1-1). All cells were maintained in an incubator at 37°C/5% CO_2_. Human PSCs were expanded in hES medium (Table 1) on a feeder layer of mouse embryonic fibroblasts. A 60–70% confluent 6-well plate of undifferentiated hPSC colonies was lifted from the feeder layer using collagenase (Thermo Fisher Scientific, 17104019). Colonies were transferred to a 10 cm dish containing hES medium without fibroblast growth factor on a shaker (+/− 50 rpm). On Day 3, the medium was changed to neural induction medium (Table 1) and refreshed every other day. After 7 d in suspension, EBs were collected and seeded on laminin-coated dishes [20 μg/ml (Sigma, L2020)]. On Day 15, cells were switched to NPC medium (Table 1). Cells were passaged 1:4 every week thereafter using collagenase and a cell lifter. After Passage 3, NPC cultures were purified using fluorescence-activated cell sorting (FACS; Yuan et al., 2011; Bauer et al., 2021). NPCs were detached from the culture plate using Accutase (StemCell Technologies, 07920) and resuspended into single cell solution. CD184^+^/CD44^−^/CD271^−^/CD24^+^ cells were collected using a FACSaria III (BD Biosciences) and expanded. To obtain hPSC-derived astroglia, NPCs (Passages 5–10) were passaged 1:4 using Accutase when confluent and subsequently grown in Astrocyte medium containing leukemia inhibitory factor (LIF; 10 ng/ml) and bone morphogenetic protein 4 (BMP4; 10 ng/ml) for 4 weeks (Table 1, Fig. 1*A*). Cells were grown on laminin-coated dishes [20 μg/ml (Sigma, L2020)] and passaged 1:4 when confluent. The passaging ratio was adapted to the proliferation rate that gradually slows down at later stages of differentiation. Following the 4 week differentiation period, astroglia could be maintained for at least an additional 6 weeks.

10.1523/ENEURO.0148-24.2024.f1-1 Figure 1-1 **Immunofluorescent labeling of NPCs and their derived astroglia cultures.** NPCs stain positive for Nestin (green) and SOX2 (red) and negative for MAP2 (cyan). Astroglia stain positive for GFAP (green) and S100B (red) and negative for MAP2 (cyan) (scale bar = 50 μm). Download Figure 1-1, TIF file.

**Figure 2.**
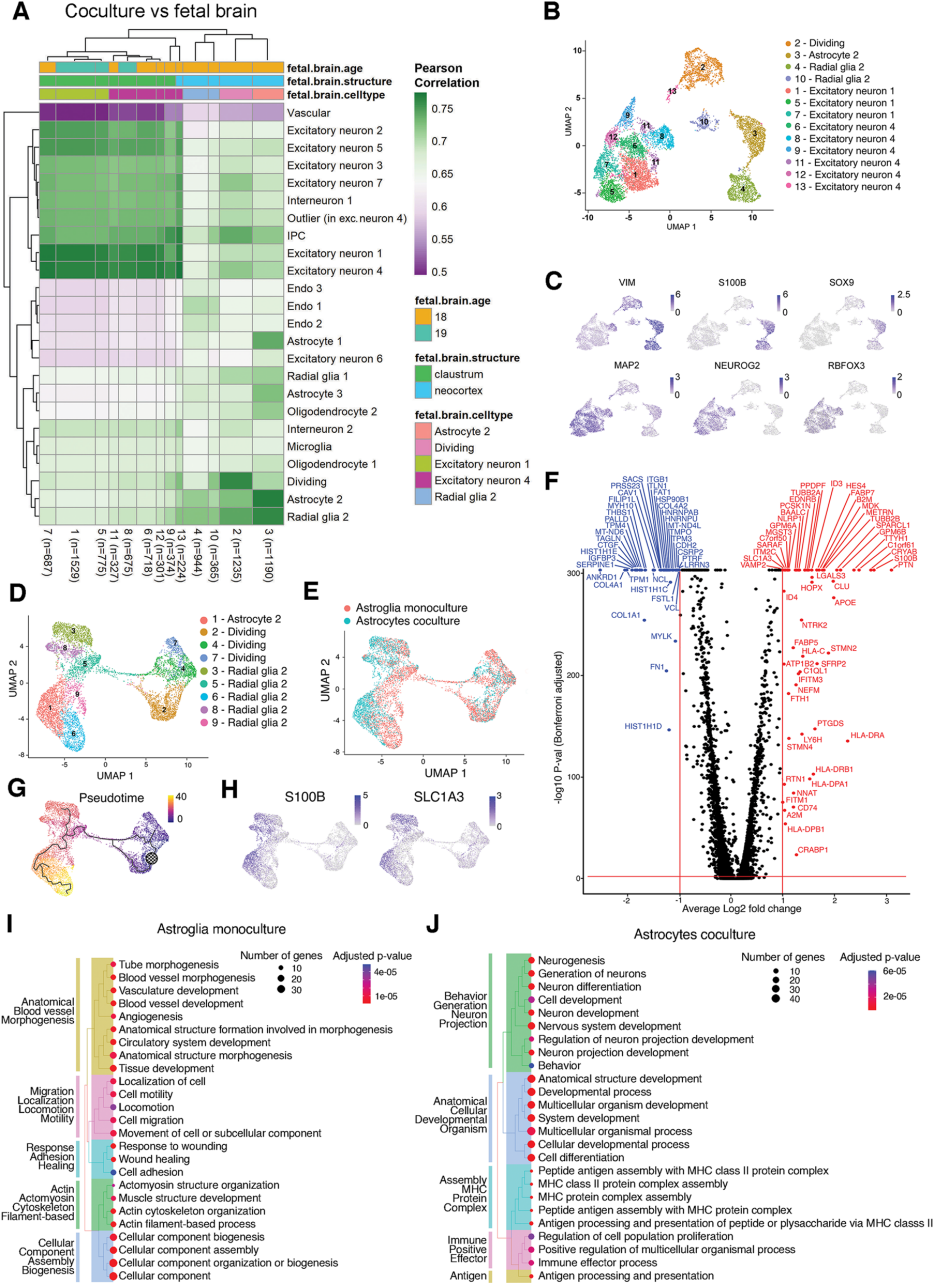
Single-cell RNA sequencing data confirms astrocyte identity and reveals transcriptional changes in coculture conditions. ***A***, Heatmap showing Pearson's correlation between scRNA-seq cell clusters of the coculture sample (iPS1) and primary fetal brain tissue (Bhaduri et al., 2021). See Extended Data Figure 2-2 for heatmaps and corresponding data for astroglial and neuronal mono-cultures. ***B***, UMAP projection of coculture sample with transferred cell type labels with the highest correlation from primary brain tissue. ***C***, Expression of genes used for selection of astrocytes (top) and neurons (bottom) from samples in the coculture sample. ***D***, UMAP projection of integrated astrocyte sample with cell type label transfer. ***E***, UMAP projection of integrated astrocyte sample with original sample identity indicated in green (coculture) or red (mono-culture). ***F***, Volcano plot of significantly up- (red) and down-regulated (blue) genes in coculture astrocytes versus mono-culture astrocytes. ***G***, Pseudotime trajectory of the integrated astrocyte sample suggests further maturation of astrocytes in the coculture sample, pseudotime indicated from blue (early) to yellow (late). ***H***, Expression of mature astrocyte markers, *S100B* and *SLC1A3*, is skewed toward cells from the coculture sample. ***I***, ***J***, Top 25 enriched GO-terms visualized in a treeplot based on DEGs in mono- (***I***) or coculture (***J***) astrocytes.

10.1523/ENEURO.0148-24.2024.f1-2 Figure 1-2 **BMP4 and LIF are required for efficient astroglia differentiation.** (**A**) Representative images of hPSC-derived NPCs (iPS1) exposed to astrocyte medium containing BMP4 (10 ng/ml) and LIF (10 ng/ml), only BMP4 (10 ng/ml), only LIF (10 ng/ml) or no additional growth factors during a 4-week period (scale bar = 50 µm). Cells were stained with GFAP, S100B and MAP2 to confirm astroglial identify and evaluate neuronal contamination. (**B**) Quantification of cells double positive for S100B and GFAP (astroglia) and MAP2 (neuronal cells). Medium containing both BMP4 and LIF is more efficient (2-way ANOVA, *P*<0.001) in differentiating NPCs towards an astroglial fate (85.39% ± 2.94 (BMP4 and LIF), 19.80% ± 1.20 (BMP4), 26.18 % ± 3.97 (LIF), 29.40 % ± 5.73 (no growth factors)), medium containing only LIF gave rise to more neuronal cells (2-way ANOVA, *P*<0.01) (8.19% ± 2.27 (BMP4 and LIF), 6.50% ± 1.07 (BMP4), 24.08 % ± 3.11 (LIF), 12.07 % ± 3.30 (no growth factors)). Download Figure 1-2, TIF file.

**Figure 3.**
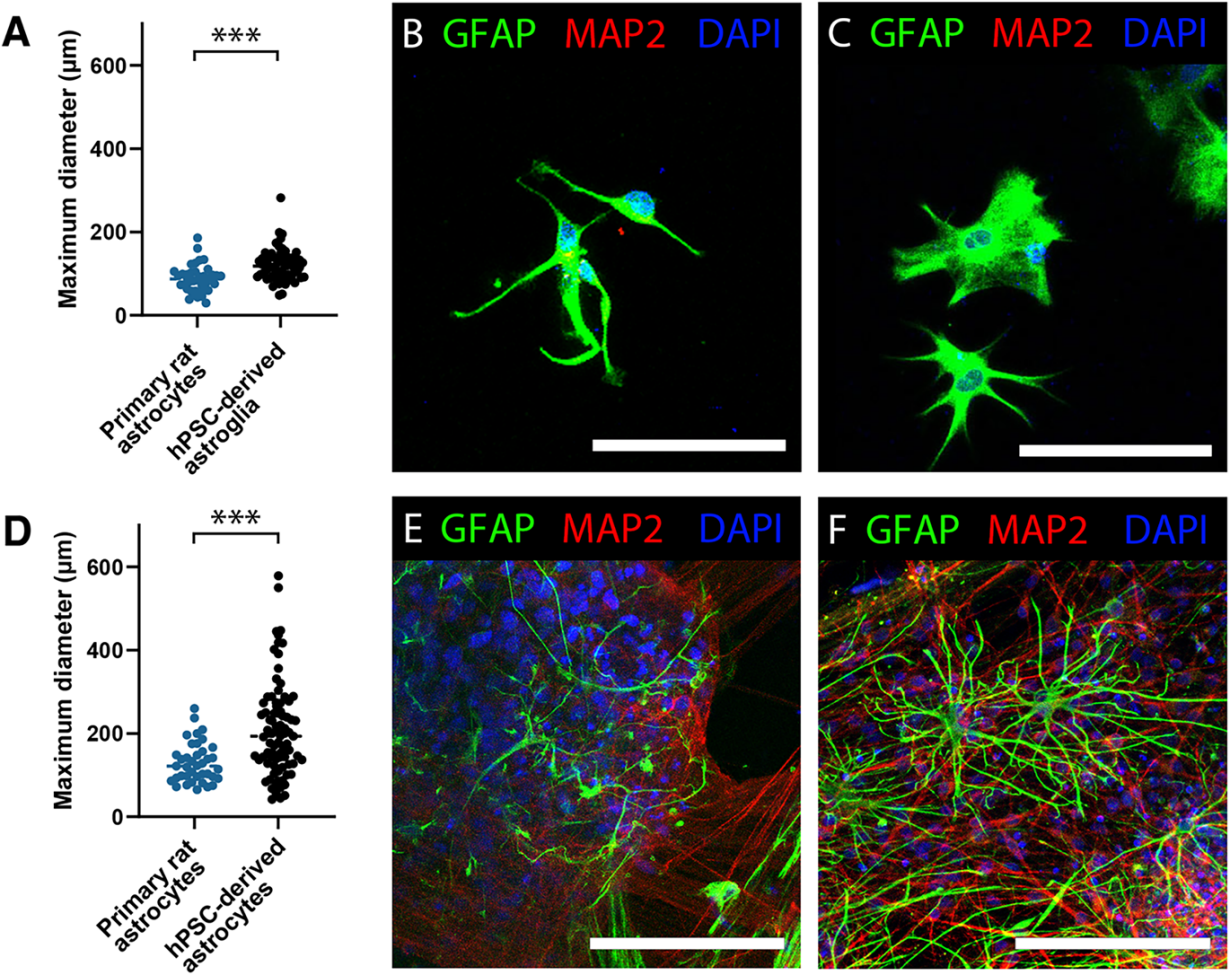
Human PSC-derived astroglia display hominid morphological characteristics in vitro. ***A***, Cell size quantification (maximum diameter) of primary rat astrocytes (blue, *n* = 38) or hPSC-derived astroglia (black, *n* = 49, iPS1) grown as a pure mono-culture. ***B***, ***C***, Representative images of primary rat astrocytes [GFAP, green (***B***) or hPSC-derived astroglia (GFAP, green; ***C***)] in a pure mono-culture (scale bars, 50 μm). ***D***, Cell size quantification (maximum diameter) of primary rat astrocytes (blue, *n* = 39) or hPSC-derived astrocytes (black, *n* = 85, iPS1) grown in a coculture with neurons. ***E***, ***F***, Representative images of primary rat astrocytes (GFAP, green; ***E***) or hPSC-derived astrocytes (GFAP, green; ***F***) in a coculture with neurons (MAP2, cyan; scale bars, 150 μm). See Extended Data Figure 3-1 for quantification of GFAP-positive cell surface of the total area in astrocyte–neuron cocultures. In vivo cell size of xenografted hPSC-derived astrocytes, postmortem mouse astrocytes, and postmortem human astrocytes is presented in Extended Data Figure 3-2.

10.1523/ENEURO.0148-24.2024.f1-3 Figure 1-3 **Principal component analysis of bulk RNA sequencing results.** PCA plot displaying all samples used for bulk RNA sequencing. Astroglia samples are depicted as circles, NPC samples as squares. Cell lines are depicted in different colors: iPS1 (purple), iPS2 (green), iPS3 (red) and ES (blue). Download Figure 1-3, TIF file.

**Figure 4.**
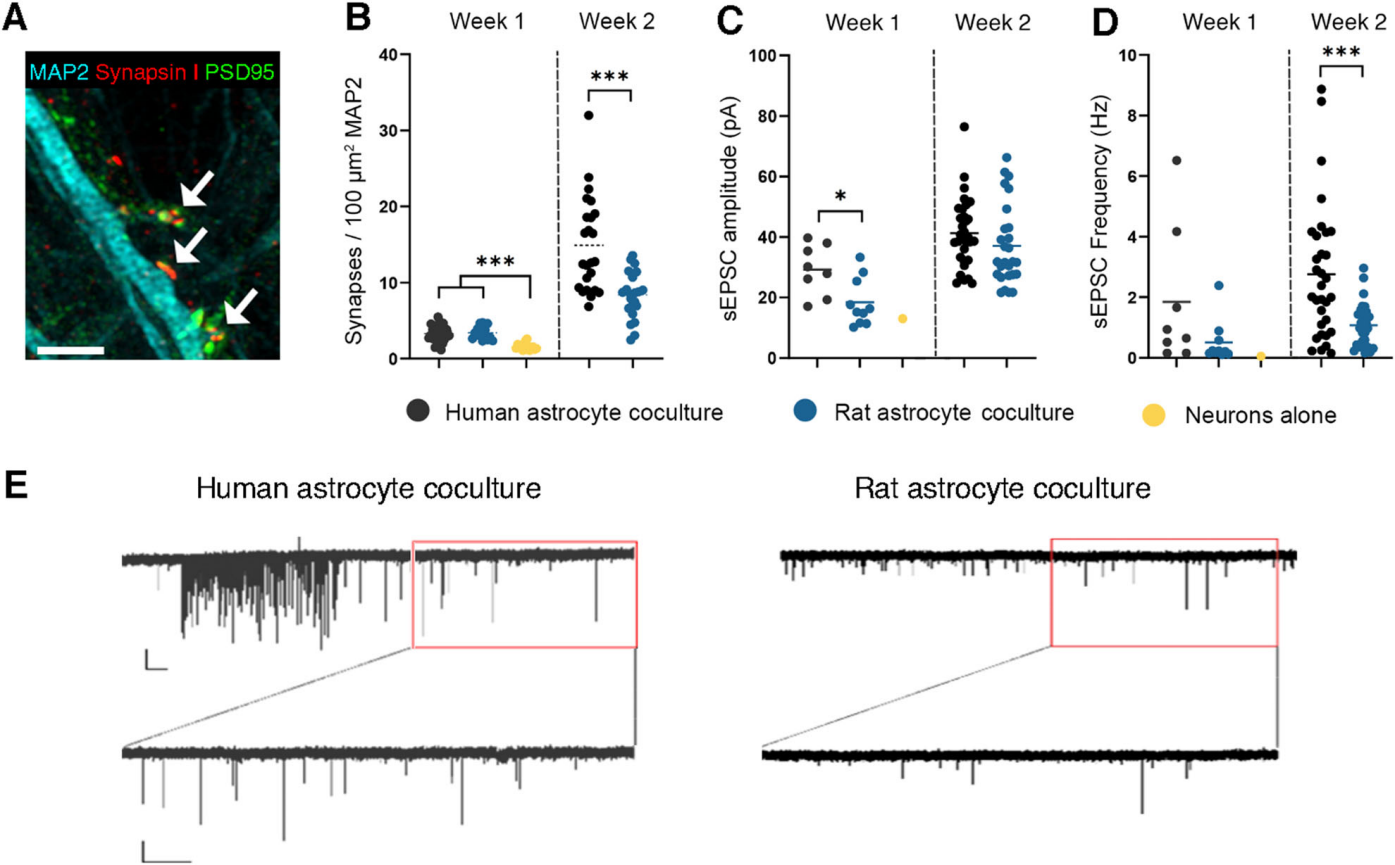
Synaptic networks are formed in cocultures of neurons with astrocytes. ***A***, Representative image of synaptic staining used for quantification, colocalization of MAP2 (cyan), synapsin I (red), and PSD95 (green) was counted as a synapse (arrows; scale bar, 3 μm). ***B***, Synapse quantification of (co)cultures at different timepoints using immunofluorescence [week 1; *n* = 35 (iPS1, human), *n* = 14 (rat) and *n* = 14 (neurons alone), week 2; *n* = 24 (iPS1, human) and *n* = 23 (rat)]. ***C***, ***D***, Quantification of spontaneous EPSC amplitude (***C***) and frequency (***D***) obtained using whole-cell patch-clamp recordings [week 1; *n* = 8 (human), *n* = 10 (rat) and *n* = 1 (neurons alone), week 2; *n* = 27 (human) and *n* = 32 (rat)]. ***E***, Representative traces of a neuron from a coculture with human (left) or rat (right) astrocytes, with a bursting event in the human coculture. Calibration, 20 pA/5 s. Additional analysis of whole-cell electrophysiological recordings is displayed in Extended Data Figure 4-1.

10.1523/ENEURO.0148-24.2024.f1-4 Figure 1-4 **Flow-cytometry quantification of astrocyte markers.** Fluorescence intensity histogram plots for iPS 1-, iPS 2- and embryonic stem cell (ES)-derived astroglia compared to primary rat astrocytes for GFAP (**A**), CD44 (**B**) and SOX9 (**C**). Download Figure 1-4, TIF file.

### Immunocytochemistry

Cells were fixed using 4% formaldehyde (FA) in PBS (Merck, 1040032500) and labeled using immunocytochemistry. Primary antibody incubation was performed overnight at 4°C. Secondary antibodies were incubated for 2 h at room temperature. Both primary and secondary antibody incubation were performed in staining buffer [0.05 M Tris, 0.9% NaCl, 0.25% gelatin, and 0.5% Triton X-100 (Sigma, T8787) in PBS, pH 7.4]. Primary antibodies and their dilutions can be found in Table 2. Secondary antibodies conjugated to Alexa-488, Alexa-647, or Cy3 were used at a dilution of 1:400 (Jackson ImmunoResearch). Nuclei were visualized using DAPI (Thermo Fisher Scientific, D1306). Samples were mounted using Mowiol 4-88 (Sigma-Aldrich, 81381) and imaged using a Zeiss LSM 800 confocal microscope.

**Table 2.** List of antibodies used

Antibody	Dilution	Manufacturer, catalog #
ALDH1L1	1:100	Abnova, H00010840-M01
Beta III Tubulin	1:100	Millipore, AB9354
CD15 V450	1:100	BD Biosciences, 561584
CD24 PE-Cy7	1:250	BD Biosciences, 561646
CD44 FITC	1:100	BD Biosciences, 560977
CD184 APC	1:250	BD Biosciences, 560936
CD271 PE	1:500	BD Biosciences, 560927
CD44	1:200	Sigma-Aldrich, SAB4700188
Human GFAP	1:200	BioLegend, 837201
GFAP	1:200	Millipore, AB5804
GFP	1:200	Abcam, ab13970
EAAT1	1:100	Sigma-Aldrich, MABN794
Human Nuclei	1:500	Millipore, MAB1281
MAP2	1:200	Synaptic Systems, 188004
PSD-95	1:100	Thermo Fisher Scientific, MA1-046
SOX9	1:500	R&D Systems, AF3075
STEM121	1:500	Takara Bio, Y40410
Synapsin I	1:200	Synaptic Systems, 106 103
S100B	1:200	Sigma-Aldrich, S2532

### Flow cytometry quantification

Cells were detached from the culture dish using Accutase (StemCell Technologies, 07920), washed, spun down, resuspended in PBS/2% FBS, and stained with a primary antibody (Table 2) on ice for 30 min. Next, cells were washed and stained using a secondary antibody (Jackson ImmunoResearch, 1:400), kept on ice for 30 min, and washed two more times. Samples were analyzed on an LSRFortessa (BD Biosciences). Secondary antibody alone was used as an isotype control.

### Bulk RNA sequencing

Total RNA was isolated from NPCs and their derived astroglia (iPS1, iPS2, iPS3, and ES cell lines, *n *= 3 per cell type) using an RNeasy mini kit (Qiagen, 74104). RNA samples were prepped using TruSeq Stranded mRNA Library kit (Illumina, 20020594). The resulting DNA libraries were sequenced according to the Illumina TruSeq Rapid v2 protocol on an Illumina HiSeq2500 sequencer. A total of 50 bp reads were generated, trimmed, and mapped against GRCh38 using HiSat2 (version 2.1.0). Gene expression values were called using htseq-count (version 0.9.1). Sequencing resulted in at least 21.1 M reads per sample, with at least 16.7 M counts in the expression profile and 22.7–25.2 thousand expressed genes. Analysis was performed using a custom R script.

### Culture dissociation and single-cell RNA sequencing

Single-cell RNA sequencing experiments were performed using hPSC-derived astroglia and *Ngn2*-induced neurons derived from the iPS1 line. For each sample, four cultures were pooled and dissociated using the Papain Dissociation System (Worthington Biochemicals, LK003150) according to manufacturer's instructions. Single-cell solutions were run on a Chromium Controller, and final libraries were generated with the Chromium Next GEM Single cell 3’ reagents kit v3.1 (dual index; PN-1000268, PN-1000120 PN-1000242, 10x Genomics) according to the manufacturer's protocol. Libraries were sequenced on an Illumina Novaseq6000 system (28-10-10-90 cycles) with a target of 25,000 reads/cell.

### Single-cell RNA sequencing data analysis

Sequenced samples were processed with the 10x Genomics Cell Ranger (v4.0.0) pipeline. Raw base call files were demultiplexed, followed by alignment and filtering of reads (using STAR v2.5.1b; Dobin et al., 2013) to the human reference genome GrCH38 (v1.2.0), after which barcodes and unique molecular identifiers were counted. Count data was processed using a custom pipeline developed with Seurat (v4.1.0; Hao et al., 2021) in the R statistical programming language (v4.0.5). Code is available on GitHub (https://github.com/kushnerlab/scRNAseqR) upon request. We used the updated version of Seurat's single-cell transform (Hafemeister and Satija, 2019; ‘sctransform’) for normalization and variance stabilization. Clusters were annotated with SingleR (v1.4.1; Aran et al., 2019) by cross-referencing to a recently published database (Bhaduri et al., 2021). Pseudotime was calculated using Monocle3 (v1.0.0; Cao et al., 2019). Sample integration was done by canonical correlation analysis of each mono- and coculture sample. Highly variable genes were defined and selected for dimensionality reduction through principal component analysis (PCA). Next, the Leiden algorithm (Traag et al., 2019) was used for hierarchical clustering. Cell selection by cluster level was based on defined marker panels (VIM, S100B and SOX9 for astrocytes; MAP2, NEUROG2, and RBFOX3 for neurons). Differential expression analysis was done using DESeq2 (v1.36.0; Love et al., 2014). FGSEA (v1.22.0; Korotkevich et al., 2021) and clusterProfiler (v4.4.4; Wu et al., 2021) were used for gene set enrichment and over-representation analyses, using org.Hs.eg.db (v3.15.0; Carslon, 2019) as the human genome-wide annotation reference and enrichplot (v1.16.2; Guangchuang et al., 2023) for visualization purposes. Data is available on the UCSC Cell Browser (https://ipsc-astrocyte-neuron.cells.ucsc.edu).

### Astroglia engraftment in neonatal *Rag2*^−/−^ mice

Human iPSC-derived astroglia were xenotransplanted into immunodeficient neonatal (P1) *Rag2*^−/−^ BALB/c mice under cryoanesthesia. Roughly 5–10 × 10^4^ astroglia were delivered in a 1 μl of PBS-drop via a 1-mm-diameter pulled glass pipette into five different sites: posterior and anterior anlagen of the corpus callosum bilaterally and in the cerebellar peduncle dorsally (Windrem et al., 2008). Mice were killed between 4 and 40 weeks of age by transcardiac perfusion with saline, followed by 4% PFA. Brains were removed; left in 4% PFA for 2 h at room temperature; transferred to a 10% sucrose/phosphate buffer (PB 0.1 M), pH 7.3; and stored overnight at 4°C. Brains were embedded in 12% gelatin/10% sucrose blocks. Fixation was performed for 2 h at room temperature in a 10% PFA/30% sucrose solution. Embedded brains were stored at 4°C before being sectioned into 40 μm slices on a freezing microtome (Leica; SM2000R). Brain sections were preincubated with a blocking buffer [0.5% Triton X-100 (Sigma, T8787) and 10% normal horse serum (NHS; Thermo Fisher, 16050122) in PBS] for 1 h at room temperature. Primary antibody incubation was done for 48 h at 4°C. Secondary antibody incubation was performed for 2 h at room temperature. Both primary and secondary antibody incubations were performed in a staining buffer (2% NHS and 0.5% Triton X-100 in PBS). Samples were mounted using Mowiol 4-88 (Sigma-Aldrich, 81381) and imaged using a Zeiss LSM 800 confocal microscope.

### Human brain immunocytochemistry

All procedures with human tissue were performed with the approval of the Medical Ethical Committee of the Erasmus MC Rotterdam, including written informed consent of all subjects for brain donation in accordance with Dutch license procedures and the Declaration of Helsinki. Fresh-frozen postmortem tissue blocks containing the middle frontal gyrus (BA9) from three donors [61 (female), 79 (male), and 81 (female) years old] were obtained from the Erasmus MC Department of Pathology. Donors were confirmed to have no past medical history of any known psychiatric or neurologic illness, with additional confirmation of the absence of clinical neuropathology by autopsy examination (Amin et al., 2018). Tissue blocks were postfixed for 7 d in 4% paraformaldehyde (0.1 M phosphate buffer), pH 7.3, at 4°C. Tissue was subsequently transferred to 10% sucrose (0.1 M phosphate buffer), pH 7.3, and stored overnight at 4°C. Embedding was performed in 12% gelatin/10% sucrose, with fixation in 10% paraformaldehyde/30% sucrose solution for 4 h at room temperature and overnight immersion in 30% sucrose at 4°C. Serial 40 μm sections were collected along the rostro-caudal axis using a freezing microtome (Leica; SM2000R) and stored at −20°C in a solution containing 37.5% ethylene glycol (Avantor, 9300), 37.5% glycerol (VWR Chemicals, 24 386.298) and 25% 0.1 M phosphate buffer. Free-floating sections were washed thoroughly with PBS before being incubated in sodium citrate (10 mM) at 80°C for 45 min and rinsed with PBS. Sections were preincubated with a blocking PBS buffer containing 1% Triton X-100 and 5% bovine serum albumin for 1 h at room temperature. Primary antibody labeling was performed in PBS buffer containing 1% Triton X-100 and 1% BSA for 72 h at 4°C. Following primary antibody labeling, sections were washed with PBS and then incubated with corresponding Alexa-conjugated secondary antibodies and cyanine dyes (1:400, Braunschweig Chemicals) in PBS buffer containing 1% Triton X-100 and 1% BSA for 4 h at room temperature. Nuclear staining was performed using DAPI (1:10,000, Thermo Fisher Scientific). Images were acquired using a Zeiss LSM 800 confocal microscope.

### Astrocyte size quantifications

Maximum projection images of 40 μm brain slices or in vitro cultures were used to determine cell size by drawing regions of interest (ROIs) around typical protoplasmic astrocytes and calculating maximum diameter with the Fiji module of NIH ImageJ. Following xenotransplantation into the murine brain, astrocytes were identified from tissue sections using a combination of antibodies (Table 2): (human) GFAP, STEM121, and human nuclear antigen (hNA). Neighboring mouse astrocytes and xenotransplanted human iPSC-derived astrocytes were analyzed in mice of different ages (4–40 weeks). Human astrocyte size was also quantified in the middle frontal gyrus of postmortem tissue from all three donors [age 61 (*n* = 9), 79 (*n* = 8), and 81 (*n* = 11)]. In vitro astrocyte size was quantified in cultures containing either primary rat astrocytes or human iPSC-derived astrocytes, from either pure mono-cultures or in coculture with *Ngn2*-induced neurons, as indicated. Astrocyte surface area was determined by thresholding for GFAP using the Fiji module of NIH ImageJ and calculating the percentage GFAP-positive cell surface of the total surface area.

### Coculture with iCell GlutaNeurons

Human PSC-derived astrocytes or rat astrocytes (ScienCell, SCCR1800) were grown in coculture with iCell GlutaNeurons (Cellular Dynamics, R1034). Astrocytes and neurons were plated in a 1:2 ratio on a 24-well multiwell microelectrode array (MEA) plate (Multi Channel Systems, 24W300-30G-288) or on coverslips, coated with poly-ʟ-ornithine (Sigma-Aldrich, P4957) and 50 μg/ml laminin (Sigma-Aldrich, L2020). Cocultures were maintained in iCell medium (Table 1) 37°C/5% CO_2_ for up to 4 weeks.

### Coculture with *Ngn2*-neurons

Human iPSCs were directly differentiated into excitatory cortical layer 2/3 neurons by forcibly overexpressing the neuronal determinant *Neurogenin 2* (*Ngn2*; Zhang et al., 2013; Frega et al., 2017). On Day 0, hiPSCs containing an integrated *Ngn2* cassette under a TET-controlled promotor were passaged on a 1:100 Matrigel coating (Sigma-Aldrich, CLS356231) and grown in StemFlex (A3349401, Thermo Fisher Scientific) containing 4 µg/ml doxycycline (D5207, Sigma-Aldrich) and 1:100 RevitaCell (A2644501, Thermo Fisher Scientific). The following day, medium was switched to *Ngn2* Day 1 medium (Table 1). To support neuronal maturation, hPSC-derived astrocytes were added to the culture on Day 2 in a 1:2 astrocyte:neuron ratio. On Day 3, the medium was changed to *Ngn2* medium (Table 1). From Day 5 onward, half of the medium was refreshed three times per week. Cocultures were kept at 37°C/5% CO_2_ throughout the entire differentiation process.

### Coculture with *Ngn2* neurons in independent laboratory

On Day 0, hiPSCs containing an integrated *Ngn2* cassette under a TET-controlled promotor were passaged on a 1:100 Matrigel coating (Sigma-Aldrich, CLS356231) and grown in E8 medium (A1517001, Thermo Fisher Scientific) containing 4 µg/ml doxycycline (D5207, Sigma-Aldrich) and 10 µM ROCK inhibitor (Y0503, Sigma-Aldrich). The following day medium was switched to *Ngn2* Day 1 medium (Table 1). To support neuronal maturation, hPSC-derived astrocytes or freshly prepared rat astrocytes were added to the culture on Day 2 in a 1:1 ratio. On Day 3, the medium was changed to *Ngn2* medium (Table 1) and cytosine β-d-arabinofuranoside (Ara-C; 2 µM; Sigma-Aldrich, C1768) was added once to remove proliferating cells from the culture, facilitating long-term recordings of the cultures. From Day 5 onward, half of the medium was refreshed three times per week. Culture medium was additionally supplemented with 2.5% FBS (Sigma-Aldrich, F2442) to support astrocyte viability from Day 10 onward. Cocultures were kept at 37°C/5% CO_2_ throughout the entire differentiation process.

### Microelectrode array recordings

Spontaneous electrophysiological activity of iCell GlutaNeurons with or without astrocytes [hPSC-derived or primary rat (ScienCell, SCCR1800)] was recorded using a multiwell MEA system (Multi Channel Systems). Plates were kept at 37°C and maintained at 5% CO_2_. Plates were equilibrated to the chamber for 10 min and recorded for an additional 10 min. The signal was sampled at 10 kHz, filtered with a high-pass Butterworth filter with 100 Hz cutoff, and a low-pass fourth-order Butterworth filter with 3,500 Hz cutoff. The noise threshold was set at +/−4.5 standard deviations. Recordings were analyzed off-line using Multiwell-Analyzer (Multi Channel Systems) and SPYCODE (Bologna et al., 2010).

### Electrophysiological recordings

iCell GlutaNeurons (Cellular Dynamics, R1034) were grown in a coculture with primary rat (ScienCell, SCCR1800) or hPSC-derived astrocytes for 1–2 weeks, after which culture slides were transferred to the recording chamber and whole-cell patch-clamp recordings were performed as previously described (Gunhanlar et al., 2018). Briefly, cultures were equilibrated to artificial cerebrospinal fluid (ACSF). In the recording chamber, slides were continuously perfused with ACSF at 1.5–2 ml/min, saturated with 95% O_2_/5% CO_2_ and maintained at 20–22°C. Recordings were performed with borosilicate glass recording micropipettes (3–6 MΩ). Data were acquired at 10 kHz using an Axon MultiClamp 700B amplifier (Molecular Devices), filtered at 3 kHz, and analyzed using pClamp 10.1 (Molecular Devices). Current-clamp recordings were performed at a holding potential of −70 mV. Intrinsic membrane properties were analyzed using a series of hyperpolarizing and depolarizing square wave currents (500 ms duration, 1 s interstimulus interval) in 5 pA steps, ranging from −30 to +30 pA. Data analysis was performed using a custom-designed script in Igor Pro-8.0 (WaveMetrics). Input resistance was calculated from the first two hyperpolarizing steps. Active properties were extracted from the first depolarizing step resulting in AP firing. AP threshold was defined by the moment at which the second derivative of the voltage exceeded the baseline. AP amplitude was measured from threshold. Neurons were categorized as “firing” if they were capable of firing three or more mature APs without significant accommodation during a depolarizing current step. Voltage-clamp recordings were performed at a holding potential of −80 mV. Synaptic events were detected using Mini Analysis Program (Synaptosoft). Bursting activity was defined as a period longer than 5 s with a frequency of >20 Hz, followed by a return to baseline.

### Astrocyte marker and synapse quantification

Following a 4 week differentiation period from NPCs to astroglia, cultures were stained for MAP2, S100B, and GFAP to evaluate differentiation efficiency. Cells that stained positive for S100B and GFAP and negative for MAP2 were considered astroglia. In order to evaluate the necessity of adding both BMP4 and LIF to the differentiation medium, we exposed NPCs to astrocyte medium containing both BMP4 (10 ng/ml) and LIF (10 ng/ml; *n* = 8), only BMP4 (10 ng/ml; *n* = 8) or LIF (10 ng/ml; *n* = 9), or no additional growth factors (*n* = 7) during a 4 week period. To quantify synapse density, (co)cultures were imaged 1 and 2 weeks after plating in different compositions: iCell GlutaNeurons (Cellular Dynamics, R1034) alone (1 week old, *n* = 14), cocultured with rat astrocytes (ScienCell, SCCR1800; 1 week old, *n* = 14, 2 weeks old, *n* = 23) or cocultured with hPSC-derived astrocytes (1 week old, *n* = 35, 2 weeks old, *n* = 24). Cultures were stained for synapsin I, PSD95, and MAP2 (Table 2). Multiple (3–5) images were taken per coverslip. Synapses were counted as puncta based on colocalization of PSD95, synapsin, and MAP2 after thresholding for MAP2 staining using the Fiji module of NIH ImageJ and normalized to total MAP2 surface area per image (Fig. 4*A*).

## Results

### Differentiation of human forebrain-patterned NPCs to astroglia

Forebrain-patterned NPCs were generated from three iPSC lines (iPS1-3) and an ES line as previously described (Gunhanlar et al., 2018) with modifications. For all four lines, NPCs expressed SOX2 and Nestin, while dendritic marker MAP2 was rarely detected, confirming the progenitor status of the NPCs (Extended Data Fig. 1-1). NPCs were cryopreserved and thawed in NPC medium at the start of a differentiation round. When confluent, cells were passaged 1:4 into astrocyte medium containing leukemia inhibitory factor (LIF) and bone morphogenetic protein 4 (BMP4) and grown for an additional 4 weeks (Fig. 1*A*; Materials and Methods). The resulting astroglia expressed the canonical markers GFAP, S100B, SOX9, and CD44 and were negative for neuronal markers such as β-tubulin and MAP2. A subset of astroglia also expressed the more mature astrocyte marker ALDH1L1 (Fig. 1*B–E*, Extended Data Fig. 1-1). Astrocyte medium without BMP4 and/or LIF failed to efficiently induce astrogenesis (Extended Data Fig. 1-2). Medium containing both BMP4 and LIF is more efficient (two-way ANOVA; *p *< 0.001) in differentiating NPCs toward an astroglial fate [85.39% ± 2.94 (BMP4 and LIF), 19.80% ± 1.20 (BMP4), 26.18% ± 3.97 (LIF), 29.40% ± 5.73 (no growth factors)], and medium containing only LIF gave rise to more neuronal cells [two-way ANOVA, *p *< 0.01; 8.19% ± 2.27 (BMP4 and LIF), 6.50% ± 1.07 (BMP4), 24.08% ± 3.11 (LIF), 12.07% ± 3.30 (no growth factors)].

To further characterize the hPSC-derived astroglia, and compare their expression profile to the parental NPCs, we performed bulk RNA sequencing of three independent differentiation batches of each of the four hPSC lines. Hierarchical clustering based on Euclidean distance using variance stabilizing transformed (VST) counts for established cell type-specific markers confirmed the distinction between NPC and astroglia samples and batch-to-batch reproducibility (Fig. 1*F*). PCA reveals that PCA 1 separates the samples on cell type (Extended Data Fig. 1-3). Canonical astrocyte genes, e.g., *GFAP*, *AQP4*, and *S100B*, were robustly upregulated in astroglia samples compared with NPC samples, while neuronal genes, e.g., *MAP2*, *TUBB3*, and *DCX*, were downregulated.

Flow cytometry based on GFAP, CD44, and SOX9 immunolabeling exhibited a similar geometric mean fluorescence intensity and cellular purity between hPSC-derived and primary rat astrocytes (Fig. 1*G–I*, Extended Data Fig. 1-4). These results together demonstrate that 4 week differentiation of NPCs in medium containing LIF and BMP4 is sufficient to obtain a relatively pure and homogenous population of hPSC-derived astroglia.

### Single-cell RNA sequencing confirms astroglial identity and reveals further specification of astrocytes in neuronal coculture

During brain development, astrocyte maturation is associated with profound transcriptional changes and mutually coregulated with neuronal maturation (Zhang et al., 2016; Shan et al., 2021). In order to gain insight into the transcriptional profile and cell type identity of hPSC-derived astroglia and the transcriptomic impact of neuronal coculture on these cells, we performed single-cell RNA sequencing (scRNA seq) in three independent conditions: (1) astroglia mono-culture, (2) neuronal mono-culture, and (3) coculture of astrocytes and neurons. As expected, the neuron and astroglia samples showed robust expression of their corresponding cell type-specific markers [e.g., *VIM* and *FABP7* (astroglia); *MAP2* and *NEUROG2* (neurons); Extended Data Fig. 2-1*A,B*].

For unbiased assignment of cell type identity to clusters in our samples, we calculated Pearson’s correlation coefficient between the individual clusters in our samples and the cell type-annotated clusters at different developmental stages (gestational weeks 14–25) from a recently published scRNAseq dataset of the developing human brain (Bhaduri et al., 2021). Using this dataset, we transferred cell type and developmental age labels to clusters in our scRNA sequencing samples. The transcriptional profile of both astroglia and neuronal mono-cultures most closely resembled the cerebral cortex during gestational week 18 (Extended Data Fig. 2-1*C,D*). Mono-culture hPSC-derived astroglia included “radial glia” (38%), “dividing” (52%), and “endothelial” (10%) cells (Extended Data Fig. 2-1*C,E*). Mono-culture neurons were most strongly correlated with excitatory neurons (90%), while the remaining 10% of cells had transcriptional profiles of dividing or intermediate progenitor cells (IPCs; Extended Data Fig. 2-1*D,F*). In the astrocyte–neuronal cocultures, we observed 60% “excitatory neurons,” 20% “radial glia,” 13% “astrocytes,” and 12% “dividing” cells (Fig. 2*A*,*B*). Interestingly, the cocultures yielded neurons with transcriptional profiles most strongly associated with gestational week 19—slightly older than neurons from the mono-culture condition—whereas mono- and coculture astrocytes are both most strongly associated with gestational week 18 (Fig. 2*A*, Extended Data Fig. 2-1*C,D*). Neurons most closely resembled “excitatory neurons,” both in mono- and coculture, consistent with their glutamatergic identity. In contrast, hPSC-derived astroglia underwent a profound transcriptional adaptation in neuronal coculture. Compared with astrocyte mono-culture, we observed fewer “dividing” cells, more “radial glia,” and a cluster observed exclusively in coculture that was most strongly correlated with “astrocytes.” Notably, in the coculture sample, cluster 10 (“radial glia”) showed a strong increase in *APOE* expression (log2fold = 4.8; *p *< 0.01) compared with all other clusters (Extended Data Fig. 2-1*H*), while in the astrocyte mono-culture we did not observe a defined cluster with *APOE* expression (Extended Data Fig. 2-1*I*).

10.1523/ENEURO.0148-24.2024.f2-1 Figure 2-1 **Single-cell RNA sequencing data from astroglia and neuron mono-culture samples.** (**A**) Astroglia sample (iPS1) shows homogenous expression of known astrocyte markers, e.g. FABP7 and VIM. (**B**) *Ngn2*-neuron sample (iPS1) shows homogenous expression of known neuronal markers, e.g. MAP2 and NEUROG2. (**C**) Heatmap showing Pearson’s correlation between scRNA seq cell-clusters of the astroglia sample and primary fetal brain tissue. (**D**) Heatmap showing Pearson’s correlation between scRNA seq cell-clusters of the Ngn2-neuron sample and primary fetal brain tissue. (**E**) UMAP projection of the astroglia sample with transferred cell type labels with the highest correlation from primary human brain tissue. (F) UMAP projection of the *Ngn2*-neuron sample with transferred cell type labels with the highest correlation from primary brain tissue. (**G**) UMAP projection of integrated *Ngn2*-neuron sample with original sample identity indicated in green (monoculture) or red (coculture). (**H**, **I**) APOE expression is upregulated in astroglia under coculture conditions and mostly expressed in a single cluster (cluster 10, “Radial glia”, arrow) (**H**), in a culture with only astrocytes (**I**) APOE expression is lower. Download Figure 2-1, TIF file.

In order to further characterize the maturation and transcriptional changes of hPSC-derived astrocytes during neuronal coculture, we next sought to identify genes that were differentially expressed in astrocytes from their corresponding mono-culture and coculture conditions. We selected clusters in which >20% of cells expressed *VIM*, *S100B*, and *SOX9* to create an integrated sample with mono- and cocultured astrocytes. Similarly, we used *RBFOX3*, *MAP2*, and *NEUROG2* as markers to select neuronal clusters from mono-culture and coculture conditions to generate a corresponding integrated neuron sample (Fig. 2*C*). By retaining the original sample identity of individual cells in these integrated samples, we were able to directly compare their gene expression profiles (Fig. 2*E*, Extended Data Fig. 2-1*G*).

In the integrated astrocyte sample, some clusters showed a skewed distribution in the original sample identity. For example, clusters labeled “dividing” contained cells mainly from the mono-culture (mono-culture, 75.8%; coculture, 24.2%), and in the cluster labeled “astrocyte” most cells originated from the coculture sample (mono-culture, 16.8%; coculture, 37.2%; Fig. 2*D*,*E*; Table 3). No such skewing of clusters was observed in the integrated neuron sample (Extended Data Fig. 2-1*G*, Table 4).

**Table 3.** Cluster composition of integrated astrocyte sample

Sample Clusters	Coculture astrocytes	Mono-culture astrocytes
1 - Astrocyte 2	1,075	484
2 - Dividing	289	1,030
3 - Radial glia 2	521	548
4 - Dividing	325	704
5 - Radial glia 2	150	701
6 - Radial glia 2	126	684
7 - Dividing	85	456
8 - Radial glia 2	288	213
9 - Radial glia 2	31	338
Total cells	2,890	5,158

**Table 4.** Cluster composition of integrated neuron sample

Sample Clusters	Coculture neurons	Mono-culture Neurons
1 - Excitatory neuron 1	1,086	682
2 - Excitatory neuron 4	1,025	570
3 - Excitatory neuron 4	663	585
4 - Excitatory neuron 4	715	351
5 - Excitatory neuron 4	429	602
6 - Excitatory neuron 4	326	415
7 - Excitatory neuron 4	505	136
8 - Excitatory neuron 4	237	206
9 - Excitatory neuron 4	173	108
10 - Excitatory neuron 4	51	55
11 - Excitatory neuron 1	76	14
12 - Excitatory neuron 4	67	22
Total cells	5,353	3,746

Next, we performed differential expression analysis on the integrated samples to gain insight into the adaptations in astrocytes and neurons during coculture. Figure 2*F* displays a volcano plot of the differentially expressed genes (DEGs) of the integrated astrocyte sample with the most highly regulated genes shown in Table 5. As expected, many well-established markers of mature astrocytes were highly upregulated in hPSC-derived astrocytes from the coculture sample, e.g., *S100B* and *SLC1A3* (Fig. 2*E*,*H*). Moreover, multiple genes were found that are known to be specifically upregulated during astrocyte–neuron interactions and coordinated maturation, e.g., *SPARCL1* (Singh et al., 2022), *METRN* (Nishino et al., 2004; Jørgensen et al., 2009), and *CROC4* (Jeffrey et al., 2000), as well as known disease-associated genes such as *CRYAB* (Guo et al., 2019) and *APOE* (Lanfranco et al., 2021; Fig. 2*F*). DEG analysis in the integrated neuron sample revealed few differentially expressed genes with a log2fold > 1, demonstrating the efficacy of *Ngn2* overexpression to induce neuronal differentiation (Table 6).

**Table 5.** List of differentially expressed (log2fold > 1) genes in mono- and coculture astrocytes

Gene ID	avg_log2FC	*p*_value_adj	pct_mono	pct_co	mean_expression_mono	mean_expression_co
PTN	3.12526763	0	0.565	0.906	1.249	4.374
S100B	2.78445545	0	0.386	0.755	0.832	3.617
CRYAB	2.72380671	3.11 × 10^−265^	0.116	0.446	0.458	3.182
C1orf61	2.49290219	2.26 × 10^−159^	0.372	0.548	1.197	3.689
TTYH1	2.39371958	0	0.566	0.81	1.218	3.612
HLA-DRA	2.27095045	6.27 × 10^−204^	0.059	0.304	0.499	2.770
GPM6B	2.13263619	0	0.688	0.904	1.374	3.507
SPARCL1	2.06041655	0	0.096	0.643	0.333	2.394
APOE	1.99927081	8.29 × 10^−236^	0.092	0.383	0.231	2.230
CLU	1.9895018	0	0.67	0.86	2.284	4.273
STMN2	1.896001	0	0.035	0.373	0.080	1.976
TUBB2B	1.80850987	0	0.894	0.967	2.049	3.857
METRN	1.78190413	0	0.517	0.955	0.930	2.712
MDK	1.71405259	0	0.799	0.944	1.773	3.487
SFRP2	1.67437032	4.97 × 10^−164^	0.356	0.608	1.146	2.820
B2M	1.67037402	7.94 × 10^−231^	0.894	0.84	2.746	4.416
PTGDS	1.63240328	1.65 × 10^−299^	0.026	0.314	0.065	1.698
HLA-DRB1	1.60230617	3.01 × 10^−180^	0.055	0.28	0.372	1.975
LGALS3	1.57583782	1.51 × 10^−12^	0.542	0.473	1.181	2.757
HOPX	1.5742526	0	0.046	0.443	0.070	1.644
FABP7	1.56986694	4.64 × 10^−260^	0.834	0.901	2.719	4.289
HLA-DPA1	1.53158385	2.27 × 10^−200^	0.035	0.258	0.182	1.713
HES4	1.51028384	0	0.482	0.867	0.891	2.401
ID3	1.48419898	1.86 × 10^−253^	0.398	0.676	0.876	2.360
PPDPF	1.43624357	0	0.82	0.989	1.536	2.972
HLA-C	1.39629787	5.60 × 10^−284^	0.402	0.73	1.099	2.495
LY6H	1.3798325	2.68 × 10^−275^	0.053	0.356	0.239	1.619
NTRK2	1.37159335	0	0.2	0.634	0.613	1.984
C1QL1	1.34285	0	0.043	0.41	0.073	1.416
TUBB2A	1.32965659	0	0.739	0.879	1.529	2.859
EDNRB	1.31242052	0	0.21	0.623	0.349	1.661
IFITM3	1.31162662	9.27 × 10^−51^	0.814	0.81	2.272	3.584
PCSK1N	1.27739151	5.85 × 10^−222^	0.429	0.664	0.807	2.084
CRABP1	1.27257082	3.31 × 10^−43^	0.12	0.239	0.835	2.107
NEFM	1.26398985	3.28 × 10^−42^	0.204	0.318	0.380	1.644
BAALC	1.25243862	0	0.186	0.67	0.338	1.590
NNAT	1.21584394	0	0.001	0.326	0.001	1.217
CD74	1.21360419	1.88 × 10^−158^	0.057	0.268	0.369	1.583
FABP5	1.2092159	1.42 × 10^−128^	0.439	0.644	0.951	2.161
STMN4	1.12504838	0	0.011	0.404	0.021	1.146
NLRP1	1.12484579	0	0.37	0.761	0.657	1.782
FTH1	1.11799685	0	1	1	4.709	5.827
GPM6A	1.10542465	0	0.22	0.617	0.388	1.493
MGST3	1.09379278	0	0.749	0.934	1.376	2.470
C7orf50	1.05799363	0	0.476	0.898	0.765	1.823
HLA-DPB1	1.05573829	1.83 × 10^−159^	0.035	0.227	0.161	1.217
SARAF	1.04571207	0	0.786	0.888	1.520	2.566
ITM2C	1.04499382	0	0.318	0.735	0.509	1.554
SLC1A3	1.04277099	1.48 × 10^−277^	0.405	0.719	0.774	1.817
A2M	1.04104507	5.23 × 10^−42^	0.262	0.37	0.825	1.866
RTN1	1.03507679	3.30 × 10^−248^	0.043	0.31	0.075	1.110
ATP1B2	1.02804356	0	0.015	0.517	0.024	1.052
ID4	1.02743718	3.54 × 10^−167^	0.43	0.647	0.821	1.849
VAMP2	1.00520525	0	0.279	0.69	0.428	1.433
IFITM1	1.00462946	6.85 × 10^−186^	0.051	0.277	0.148	1.153
VCL	−1.0171318	0	0.821	0.517	1.987	0.970
FSTL1	−1.0179856	1.50 × 10^−250^	0.782	0.543	1.975	0.957
LRRN3	−1.0240522	0	0.648	0.226	1.381	0.357
SMS	−1.026073	0	0.838	0.561	1.943	0.917
PMEPA1	−1.0488925	6.27 × 10^−207^	0.584	0.283	1.526	0.478
MT-ND5	−1.0491376	0	0.999	0.963	4.097	3.048
YWHAZ	−1.0531622	0	0.992	0.885	2.795	1.742
MYH9	−1.0553458	1.26 × 10^−288^	0.662	0.294	1.556	0.500
PTRF	−1.0588808	8.25 × 10^−267^	0.632	0.268	1.567	0.508
CSRP2	−1.0641888	0	0.931	0.654	2.537	1.473
CDH2	−1.0663212	0	0.984	0.806	2.637	1.570
TPM3	−1.0851146	0	0.965	0.754	2.382	1.297
MYLK	−1.0883518	3.01 × 10^−165^	0.418	0.13	1.417	0.329
TMPO	−1.1292194	1.50 × 10^−278^	0.686	0.324	1.853	0.724
MT-ND4L	−1.1373424	0	0.974	0.709	2.434	1.297
HNRNPU	−1.1464712	0	0.964	0.724	2.540	1.393
HNRNPAB	−1.1471103	0	0.944	0.591	2.292	1.145
HIST1H1C	−1.1843464	4.26 × 10^−151^	0.571	0.307	1.821	0.637
COL4A2	−1.1951134	1.85 × 10^−257^	0.839	0.631	2.354	1.159
HIST1H1D	−1.2114547	3.66 × 10^−110^	0.275	0.071	1.363	0.152
HSP90B1	−1.2156583	0	0.999	0.913	3.512	2.296
FAT1	−1.2367552	0	0.915	0.597	2.208	0.971
TLN1	−1.2530446	0	0.867	0.485	2.061	0.808
FN1	−1.2629606	6.08 × 10^−110^	0.399	0.164	1.788	0.525
ITGB1	−1.2650435	0	0.953	0.721	2.684	1.419
SACS	−1.2943401	0	0.854	0.364	1.861	0.566
PRSS23	−1.3034587	0	0.871	0.424	2.267	0.964
CAV1	−1.3211834	0	0.819	0.472	2.483	1.162
FILIP1L	−1.363936	0	0.565	0.117	1.558	0.194
NCL	−1.3810779	0	0.999	0.907	3.683	2.302
MYH10	−1.5078887	0	0.922	0.561	2.447	0.939
THBS1	−1.5182433	0	0.577	0.119	1.760	0.242
PALLD	−1.6739103	0	0.981	0.644	3.158	1.484
TPM4	−1.6858015	0	0.99	0.684	3.031	1.345
COL1A1	−1.6935055	5.98 × 10^−300^	0.802	0.31	3.850	2.156
MT-ND6	−1.7485323	0	0.944	0.391	2.402	0.653
TAGLN	−1.7788166	0	0.994	0.435	5.109	3.330
CTGF	−1.8333013	0	0.859	0.343	2.874	1.041
HIST1H1E	−1.8494481	0	0.623	0.16	2.134	0.284
IGFBP3	−1.8690066	0	0.523	0.06	2.155	0.286
SERPINE1	−1.9330432	0	0.567	0.117	2.306	0.373
TPM1	−2.037208	0	0.993	0.813	4.424	2.387
COL4A1	−2.0726514	0	0.882	0.612	3.395	1.322
ANKRD1	−2.5550829	0	0.571	0.01	2.584	0.029

**Table 6.** List of differentially expressed (log2fold > 1) genes in mono- and coculture *Ngn2* neurons

Gene ID	Avg log2FC	*p* value adj	Pct co	Pct mono	Mean expression co	Mean expression mono
PCSK1N	1.774	0	0.959	0.536	3.031	1.257
IGFBP5	1.682	2.31 × 10^−96^	0.184	0.019	1.749	0.067
LY6H	1.542	0	0.785	0.04	1.608	0.066
TAC1	1.447	5.86 × 10^−116^	0.242	0.023	1.509	0.062
RTN1	1.339	0	0.977	0.785	3.119	1.781
FOS	1.300	0	0.744	0.218	1.702	0.402
NTS	1.180	1.83 × 10^−32^	0.086	0.226	1.891	0.711
EEF1A2	1.105	0	0.789	0.185	1.411	0.306
SNCA	1.065	0	0.694	0.225	1.471	0.406
TUBB2A	1.014	0	0.979	0.807	2.519	1.505
SST	−1.066	6.77 × 10^−77^	0.035	0.141	0.460	1.526
DST	−1.067	0	0.729	0.966	1.246	2.313
MDK	−1.068	0	0.461	0.89	0.966	2.034
NEFM	−1.073	0	0.974	1	3.902	4.974
MT-ND6	−1.253	0	0.228	0.809	0.330	1.583

Cross-referencing the DEGs from the integrated astrocyte sample with those of a study (Zhang et al., 2016) that investigated transcriptional differences between primary human astrocyte progenitors and mature astrocytes revealed a high correspondence (Fisher's exact; *p *< 0.001; Table 7) between datasets, suggesting that astrocytes undergo additional maturation when grown with neurons. Pseudotime analysis of the integrated astrocyte sample confirmed this observation, in which “dividing” cells from the mono-culture sample are positioned at the beginning of the developmental trajectory and cocultured astrocytes enriched at the end (Fig. 2*D*,*E*,*G*).

**Table 7.** Top differentially expressed genes in human astrocyte progenitor cells and mature astrocytes as previously reported by Zhang et al. (2016)

Top astrocyte progenitor genes					Top mature astrocyte genes				
Gene	Expression in mono-culture astrocytes	Expression in coculture astrocytes	Log2fold change	Adjusted *p*-value	Gene	Expression in mono-culture astrocytes	Expression in coculture astrocytes	Log2fold change	Adjusted *p* value
*HIST1H2AI*	0.00	0.01	0.01	0.21	*AGXT2L1*	0.00	0.00	0.00	1.00
*HIST1H3E*	0.03	0.02	−0.02	1.00	*S100A1*	0.00	0.01	0.01	1.00
*HIST1H3B*	0.34	0.11	−0.23	>0.01	*SLC14A1*	0.00	0.00	0.00	1.00
*HIST1H1B*	0.66	0.23	−0.43	>0.01	*TMEM176A*	0.00	0.00	0.00	1.00
*PPDPF*	1.54	2.97	1.44	>0.01	*TMX2*	0.44	0.72	0.28	>0.01
*TPX2*	1.58	1.00	−0.58	>0.01	*HHATL*	0.00	0.00	0.00	1.00
*NUSAP1*	1.45	1.08	−0.37	>0.01	*PADI2*	0.01	0.00	0.01	1.00
*HIST1H2AC*	0.27	0.26	−0.03	1.00	*TLR4*	0.02	0.01	0.02	0.96
*HIST2H2AC*	0.57	0.21	−0.36	>0.01	*HSD17B6*	0.01	0.05	0.04	>0.01
*TNC*	0.34	0.38	−0.18	1.00	*CHI3L1*	0.01	0.01	0.04	1.00
*KIF15*	0.95	1.15	−0.19	>0.01	*NUDT3*	0.95	1.15	0.19	>0.01
*HIST1H1A*	0.01	0.00	0.00	1.00	*FBXO2*	0.02	0.12	0.14	>0.01
*HIST1H2BC*	0.02	0.12	−0.14	>0.01	*NTSR2*	Not detected	Not detected	-	-
*FABP5*	0.03	0.076	1.21	>0.01	*ALDH1L1*	0.03	0.076	0.07	>0.01
*DTYMK*	1.14	0.83	−0.32	>0.01	*ALDOC*	0.01	0.039	0.04	>0.01
*RAB11B*	0.08	0.24	0.16	>0.01	*SLC1A2*	0.00	0.05	0.06	>0.01
*HES6*	1.40	0.67	0.73	>0.01	*RYR3*	0.10	0.02	−0.08	>0.01
*LRIG3*	0.13	0.10	0.05	1.00	*GABRA2*	0.00	0.03	0.03	>0.01
*E2F5*	0.30	0.31	0.04	1.00	*CPE*	0.75	1.26	0.51	>0.01
*MPPED2*	0.02	0.05	0.03	>0.01	*GLUL*	0.33	0.58	0.50	>0.01

Highlighted genes are significantly (Bonferroni corrected) up- (green) or downregulated (red) in iPSC-derived astrocytes when grown in a coculture with *Ngn2* neurons.

In order to gain insight into the biological processes associated with DEGs in mono- or cocultured astrocytes, we performed a gene ontology (GO) pathway analysis based on genes that showed a log2fold change >1 or smaller than −1 when comparing astrocytes from the mono- to the coculture condition. GO term analysis revealed distinct transcriptional profiles active in mono- (Fig. 2*I*) or cocultured astrocytes (Fig. 2*J*), suggesting a switch in the primary cellular functioning of astrocytes depending on culture conditions. The top 25 (*p* value) GO terms based on the DEGs in the mono-culture astrocytes were associated with angiogenesis, migration and cell motility, cytoskeleton organization, and cellular component biogenesis. After coculture, the top 25 (*p* value) GO terms based on DEGs in astrocytes were related to neuronal development, anatomical and cellular development, and antigen presentation. Based on our GO term analysis, the transcriptional profile of astrocytes in a pure mono-culture suggests a prominent role in angiogenesis and migration, whereas the transcriptional profile of astrocytes in a coculture with neurons is associated with neuronal development and maturation and immune response.

### hPSC-derived astrocytes retain hominid morphological characteristics in vitro and following xenotransplantation into the murine brain

Pluripotent stem cells have provided a unique opportunity to study the development and physiology of human brain cell lineages. This is especially notable for astrocytes, the most highly dimorphic cell type between higher-order primates and other mammals (Oberheim et al., 2006). Compared with their rodent counterpart, human astrocytes are larger and have a more complex morphology. We sought to investigate whether our hPSC-derived astrocytes preserve this feature. In vitro, hPSC-derived astroglia are larger than primary rat astrocytes in both a pure astrocyte culture (hPSC, 119.60 μm ± 4.74; rat, 87.25 μm ± 5.37; two-tailed *t* test; *p *< 0.001; Fig. 3*A–C*) and when grown in a coculture with neurons (hPSC, 208.91 μm ± 12.25; rat, 129.54 μm ± 7.69; two-tailed *t* test; *p *< 0.001; Fig. 3*D–F*). In correspondence with this, the percentage of astrocytic GFAP-positive surface area is also increased in fully human cocultures when seeding identical cell numbers (hPSC, 23.71% ± 3.07; rat, 14.59 ± 2.44; two-tailed *t* test; *p *< 0.05; Extended Data Fig. 3-1). Interestingly, hPSC-derived astrocytes and primary rat astrocytes both adopt a larger protoplasmic morphology when grown in coculture with neurons compared with astrocytes from a mono-culture (Fig. 3*E*,*F*).

10.1523/ENEURO.0148-24.2024.f3-1 Figure 3-1 **Percentage of GFAP-positive cell surface of the total area in astrocyte-neuron cocultures.** The surface area percentage of primary rat astrocytes (n=12) and hPSC-derived astrocytes (iPS1, n=14). Download Figure 3-1, TIF file.

10.1523/ENEURO.0148-24.2024.f3-2 Figure 3-2 **Human PSC-derived astrocytes integrate in the mouse brain after neonatal xenotransplantation.** (**A**) 4 weeks after xenotransplantation hPSC-derived astrocytes (iPS3, human nuclear antigen (hNA), red) are mainly found in the subventricular zone of the lateral ventricles (scale bar = 200 μm). (**B**) Human PSC-derived astrocytes (iPS3, hNA, red) in the olfactory bulb of a 4-week-old mouse (scale bar = 50 μm). (**C**) Human PSC-derived astrocytes (iPS3, hNA, red) self-organize into astrocytic domains 8 weeks after xenotransplantation (scale bar = 300 μm). (**D**) Human PSC-derived astrocytes (iPS3, hNA, red) populate the mouse hippocampus 8 months after xenotransplantation (scale bar = 500 μm). (**E**) Human PSC-derived astrocytes (red, solid arrows) are larger and more complex compared to their rodent counterpart (green, open arrows) in an identical *in vivo* environment (scale bar = 50 μm). (**F**) Cell size quantification (maximum diameter) of postmortem mouse astrocytes (black, n = 26), postmortem human astrocytes (blue, n = 28 (3 donors, age: 61 (n=9), 79 (n=8) and 81 (n=11)) and xenotransplanted hPSC-derived astrocytes (iPS3, yellow, n = 27). Download Figure 3-2, TIF file.

Cell size quantification in an in vitro environment can be limited by the 2D environment in which cells are often maintained. It has previously been established that upon xenotransplantation, human astrocytes are larger compared with neighboring murine host astrocytes (Preman et al., 2021; Voronkov et al., 2022; Baranes et al., 2023). In order to investigate whether we could replicate this, hPSC-derived astroglia obtained using our protocol were xenotransplanted into the brains of neonatal immunodeficient *Rag2^−/−^* mice. One week after xenotransplantation, cells were mainly found near the subventricular zone (SVZ) of the lateral ventricles (Extended Data Fig. 3-2*A*). By 4 weeks after xenotransplantation, cells had migrated away via the rostral migratory stream from the SVZ and could be found in the olfactory bulb (Extended Data Fig. 3-2*B*). Eight weeks after xenotransplantation, human cells were more globally distributed throughout the brain (Extended Data Fig. 3-2*C*). Eight months after xenotransplantation, cells remained present in the midbrain, olfactory bulb, hippocampus, and cortex (Extended Data Fig. 3-1*D*). Following xenotransplantation hPSC-derived astrocytes evenly spread out through the host brain, reminiscent of astrocytic domains typically observed for endogenous astrocytes in vivo (Oberheim et al., 2006; Extended Data Fig. 3-1*C*). Moreover, we observed that xenotransplanted hPSC-derived astrocytes maintained their increased hominid cellular diameter (Oberheim et al., 2006) in the murine brain. Xenotransplanted hPSC-derived astrocytes had a larger maximum diameter (137.70 μm ± 10.3) compared with their murine counterparts (29.28 μm ± 2.26; one-way ANOVA; *p *< 0.001) and highly similar to those in the human postmortem brain (137.66 μm ± 5.48; one-way ANOVA, *p *= 0.99; Extended Data Fig. 3-2*E,F*).

### hPSC-derived astrocytes promote the formation of functional synapses

**Figure 5.**
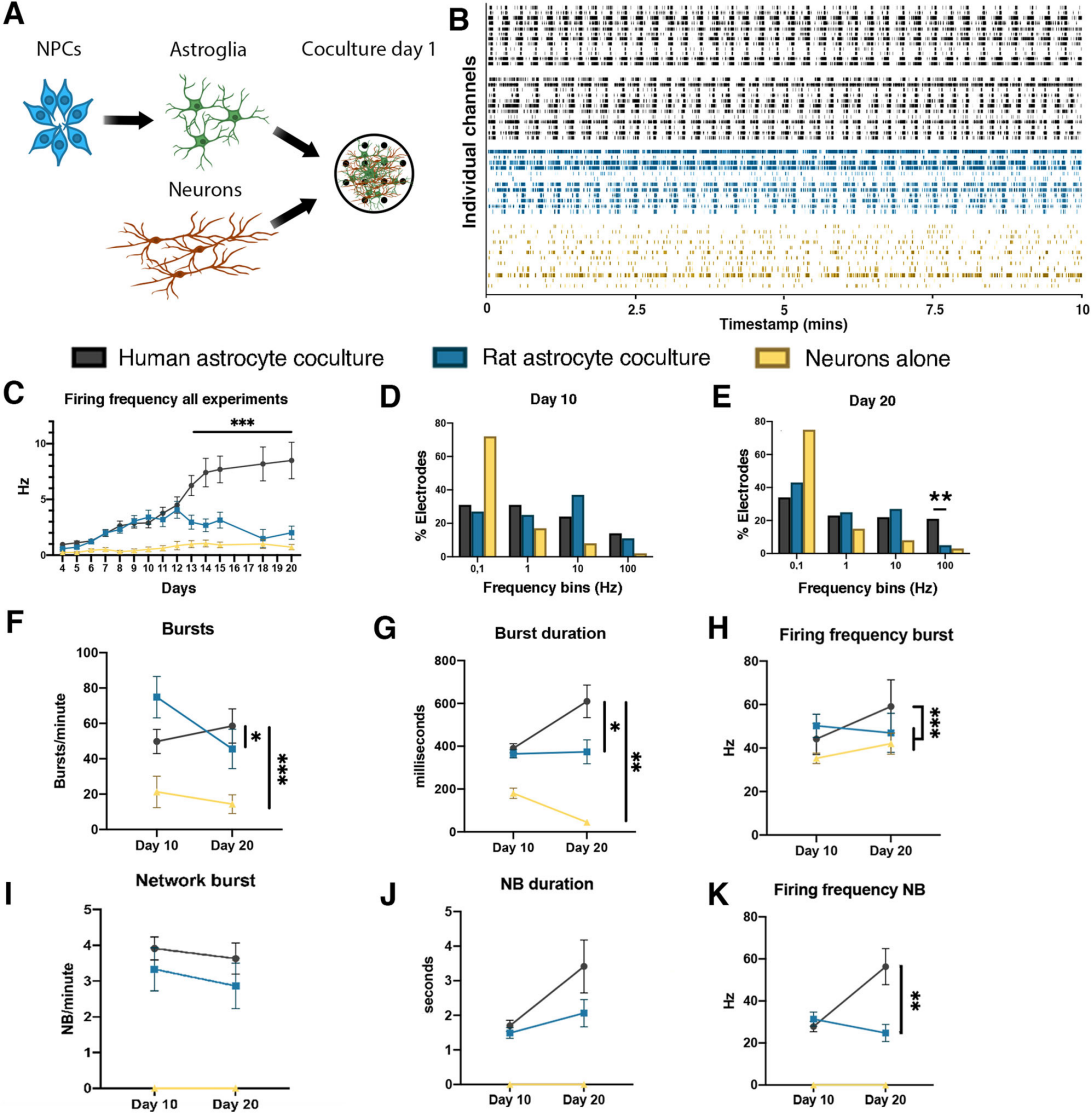
MEA recordings of (co)cultures consisting of iPSC-derived neurons with or without astrocytes. ***A***, Experimental setup of neuronal cocultures, NPCs (blue) are first differentiated to astroglia (green) and plated together with neurons (red) in a coculture [human *n* (individual wells) = 44 (iPS1 *n* = 24, iPS2 *n* = 8, iPS3 *n* = 6, ES *n* = 6), primary rat *n* = 18, neurons alone *n* = 8]. ***B***, Representative raster plots of spontaneous electrophysiological activity of individual wells in different coculture conditions, with human astrocytes (black) from different batches, rat astrocytes (blue), or neurons alone (yellow). ***C***, Mean firing frequency over time. ***D***, ***E***, Activity of all electrodes over multiple experiments presented in frequency bins 10 (***D***) and 20 (***E***) days after plating. ***F****–**H***, individual burst per minute (***F***), duration (***G***), and frequency (***H***). ***I****–**K***, Network burst per minute (***I***) and duration (***J***) are similar between rat and human astrocyte cocultures; firing frequency (***K***) is increased in human astrocyte cocultures. MEA data from an independent laboratory using hPSC-derived astrocytes is presented in Extended Data Figure 5-1.

Astrocytes are essential for proper neuronal network maturation and actively involved in the formation of synapses and establishment of network activity (Mederos et al., 2018). In order to compare species-specific astrocyte support of neuronal network maturation, we quantified synapse formation and performed MEA and whole-cell patch-clamp recordings from mono-cultured human neurons, neurons cocultured with primary rat astrocytes, or cocultured with hPSC-derived astrocytes (Figs. 4, 5).

One week after plating, we observed a greater density of synapses (Fig. 4*B*) in cocultures with hPSC-derived (3.3 ± 0.18/100 μm^2^ MAP2) or primary rat (3.4 ± 0.23/100 μm^2^ MAP2) astrocytes than in mono-cultures of neurons alone (1.5 ± 0.12/100 μm^2^ MAP2; *p *< 0.001; Fig. 4*B*). At later timepoints, we were unable to keep neurons alive without supplementing astrocytes. After 2 weeks, cocultures with hPSC-derived astrocytes had significantly more synapses (14.90 ± 1.27/100 μm^2^ MAP2) than those with rat astrocytes (8.39 ± 0.63/100 μm^2^ MAP2; *p *< 0.001; Fig. 4*B*).

10.1523/ENEURO.0148-24.2024.f4-1 Figure 4-1 **Whole-cell electrophysiological recordings of two-week old neuronal cocultures with hPSC-derived or rodent astrocytes.** (**A**, **B**) Representative traces of evoked action potentials in cultures with hPSC-derived (A) or rat (B) astrocytes. (**C**, **D**) Percentage of neurons able to fire repetitive action potentials upon current injection in cultures with hPSC-derived (C) or rat (D) astrocytes. (**E**, **F**) Percentage of neurons that receive spontaneous synaptic input in cultures with hPSC-derived (E) or rat (F) astrocytes. (**G**, **H**) Percentage of neurons that received bursts of post synaptic currents in cultures with hPSC-derived (G) or rat (H) astrocytes, this percentage was non-significantly increased in cultures with hPSC-derived astrocytes. (**I – N**) Resting membrane potential (I), input resistance (J), rheobase (K), AP threshold (L), AP amplitude (M) and AP width (N) were similar in cocultures with hPSC-derived (black) or rat (blue) astrocytes (n= 29 (hPSC, iPS1) and 30 (rat) cells). (**O**) sEPSC amplitude was similar in both conditions. (**P**) sEPSC rise time was slower in cocultures with hPSC-derived astrocytes (two-tailed t-test, *P*<0.05). (**Q**) No differences were found in the decay time of sEPSC (n= 27 (hPSC, iPS1) and 32 (rat) cells). Download Figure 4-1, TIF file.

Using whole-cell patch-clamp recordings, we observed robust spontaneous excitatory postsynaptic currents (sEPSCs) in cultures supplemented with astrocytes, while sEPSCs were nearly undetectable in cultures without astrocytes. One week after plating, sEPSC amplitude was significantly higher in cocultures with hPSC-derived astrocytes (29.21 pA ± 2.75) compared with rat astrocytes (18.42 pA ± 2.37; *p *< 0.05; Fig. 4*C*). However, by 2 weeks after plating, sEPSC amplitude was similar in cocultures with hPSC-derived (41.24 pA ± 1.97) versus rat astrocytes (37.05 pA ± 2.51; *p *= 0.19; Fig. 4*C*). The sEPSC frequency on the other hand showed a non-significant increase in 1-week-old cocultures with hPSC-derived (1.85 Hz ± 0.81) versus rat astrocytes (0.51 Hz ± 0.21; *p *= 0.09; Fig. 4*D*). By 2 weeks after plating, sEPSC frequency was significantly increased in both conditions compared with Week 1 (*p *< 0.001). Moreover, EPSC frequency was significantly higher in cocultures with hPSC-derived astrocytes (2.76 ± 0.39 Hz) compared with rat astrocytes (1.07 ± 0.14 Hz; *p *< 0.001; Fig. 4*D*). We found a non-significant increase in neurons exhibiting sEPSC burst activity in cocultures with hPSC-derived astrocytes (38.24%) versus rat astrocytes (20.69%; Fisher's exact; *p *= 0.17; Extended Data Fig. 4-1*G,H*). Moreover, EPSC rise time was slower in cocultures with hPSC-derived astrocytes (1.76 ms ± 0.08) versus rat astrocytes (1.46 ms ± 0.09; *p *< 0.05; Extended Data Fig. 4-1*P*). We observed no statistically significant differences in the intrinsic properties of neurons across the coculture conditions (Extended Data Fig. 4-1).

### Formation of high-frequency network activity in hPSC-derived astrocyte and neuron cocultures

The developmental time course of network activity was further studied using MEA recordings (Fig. 5*A*). Raster plots showing representative activity for individual wells are shown in Figure 5*B*. Neurons cocultured with astrocytes exhibited increased activity compared with neuronal mono-cultures, beginning 5 d after plating. This difference became even more pronounced over time, reaching a plateau after 2 weeks of activity. Notably, the firing rate was higher in cocultures with hPSC-derived versus primary rat astrocytes, beginning 12 d after establishment of the cultures (two-way ANOVA; *p *< 0.005; Fig. 5*C*). Twenty days after plating, 21% of the electrodes in hPSC-derived astrocyte cocultures exhibited a firing rate >100 Hz. In contrast, for cocultures with rat astrocytes, only 5% of the electrodes recorded firing frequencies >100 Hz (Fisher's exact; *p *< 0.01; Fig. 5*D*,*E*). In addition to overall firing rates, we also quantified individual burst activity (Fig. 5*F–H*). At Day 20, bursts occurred more frequently in neuronal cocultures with hPSC-derived astrocytes (58.56 bursts/min ± 9.66) compared with rat cocultures (45.56 bursts/min ± 11.12; *p *< 0.05) or without astrocytes (14.33 bursts/min ± 5.30; *p *< 0.001; Fig. 5*F*). Bursts were also of a longer mean duration in neuronal cocultures with hPSC-derived astrocytes (609.93 ms ± 76.00) compared with rat astrocytes (373.94 ms ± 55.57; *p *< 0.05) or without astrocytes (44.84 ms ± 7.29; *p *< 0.01; Fig. 5*G*) and exhibited a higher within-burst firing frequency [hPSC-derived (59.13 Hz ± 2.20), rat (46.98 Hz ± 2.32; *p *< 0.001), without astrocytes (42.01 Hz ± 1.97; *p *< 0.005); Fig. 5*H*].

10.1523/ENEURO.0148-24.2024.f5-1 Figure 5-1 **Implementation of hPSC-derived astrocytes in an independent laboratory.** (**A**) Experimental setup of neuronal coculture. Human iPSCs are plated together with astrocytes in a coculture and *Ngn2* overexpression is induced in iPSCs using doxycycline to initiate neuronal differentiation. (**B** - **D**) Analysis of *Ngn2*-neuronal cocultured with hPSC-derived astrocytes (iPS1, n = 10) or primary rat astrocytes (n = 9). Mean firing frequency within NBs (B) and NB rate per minute (C) is increased in hPSC-derived astrocyte cocultures, while network burst duration is similar across conditions (D). Download Figure 5-1, TIF file.

Network bursts (NBs) were defined as simultaneous bursts in at least 50% of all electrodes in a well. NBs were only detected in cocultures, with no detectable NBs in neuronal cultures without astrocytes. At Day 20, cocultures using hPSC-derived and rat astrocytes NBs were similar in frequency [3.63 ± 0.44 min^−1^ (hPSC-derived), 2.86 ± 0.64 min^−1^ (rat); (*p *= 0.19) Fig. 5*I*] and duration [3.4 ± 0.77 s (hPSC-derived), 2.1 ± 0.39 s (rat); (*p *= 0.10) Fig. 5*J*]. However, at Day 20 firing frequency within NBs was higher in cocultures with hPSC-derived astrocytes (56.31 ± 8.56 Hz) compared with rat (24.77 ± 4.04 Hz; *p *< 0.01; Fig. 5*K*).

In an effort to evaluate the robustness of our findings, an independent laboratory (Nijmegen) compared their standardized workflow for MEA-based recordings of cocultures of human *Ngn2*-induced neurons with rat astrocytes by substituting with hPSC-derived astrocytes (Extended Data Fig. 5-1*A*; Frega et al., 2019; Mossink et al., 2021; S. Wang et al., 2022). Neuronal firing frequency was significantly increased in cocultures with hPSC-derived versus rodent astrocytes (two-way ANOVA; *p *< 0.001; Extended Data Fig. 5-1*B*). Furthermore, hPSC-derived astrocyte cocultures showed an increased NB frequency (*p *< 0.005; Extended Data Fig. 5-1*C*) and similar NB duration (*p *= 0.70; Extended Data Fig. 5-1*D*).

## Discussion

Astrocytes are essential for neuronal network development, survival, and electrophysiological maturation (Pyka et al., 2011; Clarke and Barres, 2013; Tang et al., 2013). Compared with their rodent counterpart, human astrocytes are larger and have a more complex morphology. A specific subtype of astrocyte, interlaminar astrocytes, is found exclusively in higher-order primates (Colombo et al., 1998). Primary human astrocytes have previously been shown to have functional differences with rodent astrocytes, as well (Diniz et al., 2012; Zhang et al., 2016). Accordingly, there is a widely acknowledged need for protocols to obtain functional astrocytes from human pluripotent stem cells, demonstrated by the many protocols that currently exist (Caiazzo et al., 2015; Kondo et al., 2016; Krencik et al., 2017; Sloan et al., 2017; Tcw et al., 2017; di Domenico et al., 2019; Garcia et al., 2019; Hedegaard et al., 2020; Shih et al., 2021; Jovanovic et al., 2023). By adopting existing protocols and supplementing culture media with BMP4 and LIF, we were able to efficiently establish a pure culture of functional hPSC-derived astroglia from an intermediate cryopreserved NPC stage in 28 d. By making use of an intermediate stage of NPCs that can be expanded and survives cryopreservation, human astrocyte cultures can be rapidly established while maintaining the genomic integrity of the parental hPSC line. Immunocytochemistry and RNA sequencing reveals a relatively homogenous population of cells with widespread expression of canonical astrocyte markers such as GFAP, S100B, SOX9, and CD44 (Fig. 1). Single-cell RNA sequencing shows that the transcriptional profile of these cells is most similar to primary human “radial glia,” “astrocytes,” and a group of “dividing” cells (Fig. 2*A,B*, Extended Data Fig. 2-1*C*).

BMP4 and LIF have previously been demonstrated to promote differentiation of neural stem cells to astrocytes (Mabie et al., 1997; Koblar et al., 1998; Bonaguidi et al., 2005). Canonically, growth factors from the BMP family signal via SMAD-dependent pathways (R. N. Wang et al., 2014), whereas LIF activates the JAK/STAT pathway (Bonni et al., 1997). During late embryogenesis, the binding of the STAT3–SMAD1 complex to astrocytic promotors induces further maturation of glial progenitors toward astrocytes (Nakashima et al., 1999). In vitro, treatment of murine embryonic subventricular zone progenitor cells with LIF generates proliferating GFAP^+^ astrocyte progenitor cells, after which subsequent BMP4 exposure further differentiates these cells to mature astrocytes (Bonaguidi et al., 2005). Our approach for deriving astrocytes from human pluripotent stem cells (hPSCs) leverages this signalling mechanism by first establishing hPSC-derived NPCs and then subsequently inducing astrocyte differentiation through addition of BMP4 and LIF, thereby resulting in a pure culture of proliferating hPSC-derived astroglia.

A widely adopted method that rapidly yields a pure population of hPSC-derived neurons through forcible overexpression of *Ngn2* (Zhang et al., 2013) was developed using supplementation with primary rodent astrocytes (Frega et al., 2015). Here, we demonstrate the ability to establish a fully human PSC-derived neural coculture system (Figs. 4, 5). This provides the opportunity to precisely control the cellular composition, making it possible to study the effects of cell type-specific genotypes or targeted genetic manipulations of cocultured astrocytes and/or neurons. In addition, we show that hPSC-derived astrocytes can be efficiently integrated into the existing workflow of an independent laboratory (Extended Data Fig. 5-1), emphasizing the robustness of the method.

Another advantage of the method is the relative ease of producing hPSC-derived NPCs and astroglia compared with using rodents for obtaining primary astrocytes and thereby also contributes to reducing the use of laboratory animals. Primary murine astrocyte cultures can be established within 30 d following dissociating the brain of a P0 pup (Güler et al., 2021), provided one has access to an active laboratory animal facility. Obtaining hPSC-derived NPCs can take up to 60 d depending on the protocol used; we utilize an embryoid body approach (Gunhanlar et al., 2018), while a different laboratory has used our protocol to successfully establish hPSC-derived astroglia from NPCs differentiated using a dual-SMAD inhibition approach (Gordillo-Sampedro et al., 2024). Once hPSC-derived NPCs have been established, these can be expanded and cryopreserved. By making use of an intermediate stage of NPCs, human astrocyte cultures can be rapidly established while maintaining the genomic integrity of the parental hPSC line. From the NPC stage, obtaining hPSC-derived astroglia requires a similar time investment as primary murine astrocytes, without the need for an active animal colony. If a laboratory is equipped to perform in vitro experiments, no specialized equipment is required to integrate our protocol into their workflow.

Through scRNA sequencing, we gained insight into the transcriptional profile of different populations of astroglia and observed profound changes in their profile when cocultured with neurons. Even though we detected a relatively uniform expression of GFAP, S100B, and SOX9 in pure hPSC-derived astroglia, cross-referencing our scRNAseq data to a human fetal brain database (Bhaduri et al., 2021) revealed distinct astroglial subtypes within this in vitro astrocyte population, especially when cocultured with neurons (Fig. 2*A*, Extended Data Fig. 2-1*C*). This finding highlights the importance of using an unbiased approach when assigning cell type identity to scRNA sequencing data, as relying on a small set of genes to verify cell type identity would have masked the cellular diversity in our hPSC-astroglia populations.

Human PSC-derived astroglia seem to undergo additional developmental specification when cocultured with neurons. We demonstrate that this adaptation changes the transcriptional profile of astroglia and their associated cellular subtype diversity (Fig. 2*D*). In vivo, astrocytes are traditionally divided into subtypes based on their morphology (Oberheim et al., 2012). Recent attempts have been made using scRNA sequencing techniques to further specify astrocyte subtypes (Batiuk et al., 2020; Qian et al., 2023). Here we show that it is also possible to make a distinction in vitro based on their transcriptomic profile, e.g., cluster 10 (radial glia 2, Fig. 2*B*) displays a distinct change in *APOE* expression (Extended Data Fig. 2-1*H,I*). The identification of such a specific cluster of cells provides future opportunities for studies focused on the etiology of Alzheimer's disease and highlights the importance of establishing adequate culture conditions when using hPSC-derived cultures to study genes of interest.

DEG analysis in the integrated neuron sample revealed few differentially expressed genes with a log2fold > 1 (Table 6). It has previously been established that continuous *Ngn2* overexpression is sufficient to induce transcriptionally mature neurons (Lin et al., 2021). Interestingly, we did observe significant upregulation in cocultured neurons of *FOS* (log2fold = 1.30; *p *< 0.01), an immediate early gene regulated by neuronal activity (Curran and Morgan, 1995). The increased synaptic maturation we observed in our cocultures, despite the lack of increased synaptic gene expression in the DEG analysis, could originate from the local and transient transcriptomic changes required for synaptic plasticity that might not be reflected in a culture that has reached a homeostatic equilibrium. Furthermore, the procedure of dissociation of the neural cultures for scRNA will likely result in a negative selection against local synaptic transcripts.

We demonstrate that hPSC-derived astroglia maintain morphological hominid characteristics (Oberheim et al., 2006), both in vitro and in vivo. When comparing the morphology of primary rat and hPSC-derived astroglia in vitro, we found that hPSC-astroglia are larger (Fig. 3*A*), in line with a previous report (Zhang et al., 2016) comparing primary cultures of human and rat astrocytes. Interestingly we also observe a change of in vitro morphology upon coculture, in which astrocytes are larger and more complex when grown in coculture with neurons (Fig. 3*D*). When xenotransplanted into the murine brain, this species difference persists. Human PSC-derived astrocytes are larger compared with neighboring mouse astrocytes (Extended Data Fig. 3-1*F*). These findings confirm the bidirectional interaction between neurons and astrocytes. This is in line with a previous report that tissue microenvironment is a critical driver of astrocyte diversity in vivo (Endo et al., 2022).

Moreover, we demonstrate that hPSC-derived astrocytes are able to more efficiently support the development of synapses compared with primary rat astrocytes (Fig. 4) and the maturation of neural network activity (Fig. 5). Our findings show that hiPSC-derived neurons are more active and receive more synaptic input in a coculture with hPSC-derived versus rodent astrocytes. Synapse formation is accelerated in a fully human coculture system, resulting in an increased detection of sEPSCs through whole-cell electrophysiology (Fig. 4). Using MEA recordings, we observed a higher firing frequency in cocultures with hPSC-derived astrocytes compared with primary rat astrocytes, both for individual events and within (network) bursts (Fig. 5). We were unable to detect any substantial synapse formation or neuronal activity in cultures without astrocytes and had great difficulty maintaining these cultures for prolonged periods of time, illustrating the crucial role astrocytes play in neuronal maturation and network formation.

In a coculture with human excitatory neurons, we show that both rat and hPSC-derived astrocytes promote the formation of functional synapses. Whole-cell electrophysiological recordings suggest that the increased activity of the neuron–astrocyte cocultures observed using MEAs (Fig. 5) is not the result of changes in intrinsic neuronal properties (Extended Data Fig. 4-1), but more likely due to an increased frequency of EPSCs in the absence of a change in EPSC amplitude (Fig. 4*C*,*D*). Furthermore, our findings of an increased density of synapses in cocultures with hPSC-derived astrocytes (Fig. 4*B*) are likely to underlie at least a substantial proportion of the increase in EPSC frequency. The role of astrocytes in the formation and proper functioning of synapses has long been established (Allen and Eroglu, 2017). In correspondence with previous literature (Diniz et al., 2012), this process is accelerated in a fully human coculture system. As we demonstrate that hPSC-derived astrocytes are larger and cover more surface area compared with rat astrocytes in a coculture with neurons (Extended Data Fig. 3-1), it could be that the observed increases in neuronal activity are due to an increase in the total astrocyte cellular content in a fully human coculture system. Another possibility is that hPSC-derived astrocytes express an increased number of ion channels on their membrane or secrete different amounts of synaptogenic proteins, as has been described before for primary human astrocytes (Diniz et al., 2012). Importantly, however, we acknowledge that our experimental design precludes our ability to draw any meaningful conclusions about possible evolutionary differences between human and rat astrocytes.

The astroglia in this study were established from hPSC-derived NPCs in 28 d using a combination of BMP4 and LIF. We show that the functionality of these astrocytes compares favorably with rat astrocytes in a coculture system with human neurons. Human astrocyte–neuron cocultures are more active and mature more rapidly compared with a coculture of human neurons and rat astrocytes. Taken together, our data highlight the functional advantage of using hPSC-derived versus rat astrocytes for neuronal culture. Moreover, a fully human neuron–astrocyte coculture system provides a platform with a human genomic background for investigating astrocyte function and neuronal–glial interactions.

## Synthesis

Reviewing Editor: Benjamin Deneen, Baylor College of Medicine Decisions are customarily a result of the Reviewing Editor and the peer reviewers coming together and discussing their recommendations until a consensus is reached. When revisions are invited, a fact-based synthesis statement explaining their decision and outlining what is needed to prepare a revision will be listed below. The following reviewer(s) agreed to reveal their identity: NONE. Note: If this manuscript was transferred from JNeurosci and a decision was made to accept the manuscript without peer review, a brief statement to this effect will instead be what is listed below.

Overall the reviewers find the study to potentially be of interest, however there are several major issues that must be addressed before we can further consider this manuscript. First, is the issue of differentiation status of these cells. The reviewers feel that in the absence of additional data, it's very difficult to claim that these cells are in fact actual astrocytes and more likely reflect a more immature state of the differentiation of the lineage. Accordingly, it is recommended that the framing and overarching conclusions , statements of the manuscript be revised to reflect this. Second, there are a series of important control experiments that are missing and it is essential that these experiments be conducted to shore up the core findings of this manuscript. Third, there are a series of comments related to astrocyte size, the engraftment studies, and issues pertaining to methods and phrasing that should also be amended in a revised manuscript.

## Author Response

9th May 2024 Dear Dr. Kushner:

I am pleased to inform you that your manuscript, "Human pluripotent stem cell-derived astrocyte functionality compares favourably to primary rat astrocytes," has been judged potentially suitable for publication in eNeuro, provided appropriate revisions are made. The decision was a result of the reviewers and me coming together and discussing our recommendations until a consensus was reached. I have put together a fact-based synthesis statement explaining the decision and appended it to this email.

It is important that you revise your manuscript to address the reviewers' concerns and submit a point-by-point reply to each concern. Before making a final decision, I will carefully review your revisions and responses to the reviewers.

Your revision must include the manuscript with new text indicated in a bold or highlighted font to aid the re-review of the manuscript. Separately, please provide a clean copy of the manuscript that includes the title page, which will be published if accepted. Please closely review your manuscript at this time for any final corrections in style or substance. Please consult our Revised Submission Checklist for full details on preparing your revision: https://www.eneuro.org/content/revising-finalizing-manuscript#revisions In an effort to improve rigor, transparency, and reproducibility, eNeuro strongly encourages the use of estimation statistics. If the use of estimation statistics applies to your research, you may consider including it in your revision. More details and resources can be found in the Information for Authors: https://www.eneuro.org/content/preparing-manuscript#statistics Your submission must include publication-quality figures, each in a separate EPS or TIFF (300 dpi) file. Please make sure your figures adhere to style requirements to avoid delays in manuscript processing. Detailed guidelines for figures are available in our Information for Authors: https://www.eneuro.org/content/preparing-manuscript#figures When uploading the revised manuscript, the corresponding author will need to complete the electronic License to Publish form; if not completed during submission, a link to the form will also be available on the author's home page at https://eneuro.msubmit.net.

All authors are invited to submit visually appealing, high-quality images to be used to promote their article, including on the journal homepage. Please submit your images, as well as an image caption and credit information, directly to the peer review system with your revision. Additional details on sizing and image specifications can be found in the Information for Authors: https://www.eneuro.org/content/revising-finalizing-manuscript#image Please return your revision within 3 months of this decision. When you are ready to submit your revision, you can log in using the link below and click on the manuscript number to create your revision. If you have any questions or concerns, or need more time to prepare your revision, please contact eneuro@sfn.org.

Yours sincerely, Benjamin Deneen Reviewing Editor eNeuro --------------------------------------------- Manuscript Instructions --------------------------------------------- Synthesis of Reviews:

Computational Neuroscience Model Code Accessibility Comments for Author (Required):

N/A Synthesis Statement for Author (Required):

Overall the reviewers find the study to potentially be of interest, however there are several major issues that must be addressed before we can further consider this manuscript. First, is the issue of differentiation status of these cells. The reviewers feel that in the absence of additional data, it's very difficult to claim that these cells are in fact actual astrocytes and more likely reflect a more immature state of the differentiation of the lineage. Accordingly, it is recommended that the framing and overarching conclusions, statements of the manuscript be revised to reflect this. Second, there are a series of important control experiments that are missing and it is essential that these experiments be conducted to shore up the core findings of this manuscript. Third, there are a series of comments related to astrocyte size, the engraftment studies, and issues pertaining to methods and phrasing that should also be amended in a revised manuscript.

Dear Dr. Deneen, Thank you for inviting us to submit a revised manuscript to address the concerns raised by the reviewers. We would like to express our appreciation to the reviewers for their detailed comments and were pleased to read that both reviewers deemed our work of adequate scientific rigor. In order to address their concerns, we performed additional experiments and modified the manuscript text to further clarify our conclusions.

The major concern raised by both reviewers was the cell-type identity of the cells that were used in our manuscript. To further investigate the cell-type identity of the hPSC-derived cells used in our manuscript, we have now performed single-cell RNA sequencing (scRNA seq) in three independent conditions: a) hiPSC-derived astrocyte mono-culture, b) hiPSC-derived neuronal mono-culture, and c) co-culture of hiPSC-derived astrocytes and neurons. In addition to gaining further insight into cellular identity, this approach also allowed us to further characterize the maturation and transcriptional changes induced by co-culture conditions. We observed a mixed population of glia from the astrocyte lineage in our cultures. In accordance with the scRNAseq results, we now refer to mono-culture astrocytes as 'astroglia', and when grown in the presence of neurons we refer to these cells as 'astrocytes'.

Reviewer 1 requested additional control experiments to demonstrate the efficacy of BMP4 and/or LIF alone when differentiating iPSC-derived NPCs to astrocytes. We have now included additional data (Figure 1-2) to answer this question. Reviewer 1 proposed the hypothesis that any effect we see on neuronal activity could be due to a total increase in astrocyte content in human astrocyte-neuron co-cultures, which we have now directly quantified and included as Figure 3-1.

As requested by reviewer 2 we have included a principal component analysis from the bulk RNA sequencing as Figure 1-3.

A point-by-point response to the reviewers' comments is listed below.

Reviewer 1 In the manuscript "Human pluripotent stem cell-derived astrocyte functionality compares favourably to primary rat astrocytes" the study aims to directly compare human pluripotent stem cell (hPSC)-derived astrocytes and rat primary astrocytes on their ability to support human neural network activity and maturation, using a combination of synapse staining, patch clamp, and multielectrode array recordings. This study also includes other characterizations such as gene expression and cell size measurements in vitro and in vivo after engraftments. They conclude that hPSC-derived astrocytes have similar, or increased, effects upon cocultured neurons. They state this study is impactful as their novel combined LIF+BMP4 protocol delivers functional/pure astrocytes that may replace 'gold standard' rat astrocytes for physiological studies of hPSC-neurons. Though the study's aim is clear and scientific rigor is mostly well described to support their conclusions with biological replicates, there are several issues that reduce enthusiasm for publication. For example, novelty is low, there is a lack of controls, there is a lack of citing very similar previous studies, and some experiments such as transplantation are not designed to test the main aim of the study.

We thank the reviewer for the support of our study and the opportunity to address these important concerns.

Specifically:

1. A major issue with this manuscript is the premise and the novelty of this study. They state that rodent astrocyte coculture is the gold standard for conducting functional studies of neurons. However, this premise is a bit outdated. hPSC-derived astrocytes have already been utilized for this purpose for over a decade among a large number of studies, and this has become common practice. hPSC-astrocytes are commercially available and many protocols already exist for in-house preparation. In addition, there are numerous media supplements and maturation components that can generate physiologically-active neurons with synapses in the absence of astrocyte co-culture. The authors need to either provide specific recent examples supporting that it is still unclear in the field that using hPSC-astrocytes, or media supplements, is not as good as using rodent primary astrocytes.

We greatly appreciate the Reviewer raising this concern, the Reviewer states that many protocols have been established during the past decade to create hPSC-derived astrocytes and we fully agree with this statement. Although we are not aware of a study that describes the establishment of astrocytes from NPCs in 4 weeks by removing fibroblast growth factor from the medium and supplementing with only BMP4 and LIF, our aim was not to focus on this protocol as novel per se. We believe our work is novel and valuable to the field due to the extensive functional characterization of astrocyte-neuron co-cultures and the direct comparison to primary rodent astrocytes.

We have provided additional text and recent literature references (Wang et al., 2022; Bullmann et al., 2024), including one recent review (Hulme et al., 2022), in which mature electrophysiological data could only be obtained from Ngn2-neurons in a co-culture with rodent astrocytes (page 4). Studies utilizing Ngn2-neurons generally use primary rodent astrocytes in their co-culture models, as the general consensus in the field remains that hPSC-derived astrocytes are not suitable for establishing similarly robust functional neuronal activity in vitro.

In order to address the concern regarding previous literature utilizing hPSC-derived astrocytes, we have now included additional high-quality literature that makes use of hPSC-derived astrocytes in the Introduction section (Caiazzo et al., 2015; Kondo et al., 2016; Krencik et al., 2017; Sloan et al., 2017; Tcw et al., 2017; di Domenico et al., 2019; Jovanovic et al., 2023) (page 4). Astrocytes in these studies were often characterized by comparing transcriptomic data to primary rodent astrocytes. In addition to this we have performed an extensive comparison of the electrophysiological properties of hPSC-derived neurons in a co-culture with hPSC-derived or primary rat astrocytes, adding to the novelty of our manuscript.

2. The hPSC-astrocyte protocol is not clear and a bit misleading. It is standard to report on the total length of time it takes to generate astrocytes from the hPSC stage. However, here they emphasize in the abstract and elsewhere that cells are matured 28 days from NPCs, yet the age of NPCs are not clearly defined and NPC purification was necessary. The total age of the astrocytes during experimentation, and the use of purification, should be clearly noted in the abstract and elsewhere.

We would thank the Reviewer for pointing this out and have now revised the manuscript text to clarify the timing and purification of NPCs (page 7). Protocols to establish hPSC-derived NPCs through an embryoid body stage (our approach) or dual-SMAD inhibition have become common practice in the field over the past decade. These NPCs are often expanded and cryopreserved before being used in experiments, which prevents prolonged culturing of hPSCs and consequent protection of genomic integrity of the parental hPSC-line.

The FACS purification step is not strictly necessary to generate astrocytes. We have previously differentiated an unsorted NPC-line to astrocytes using our protocol, see the image below (Figure 1). When trying to compare different hPSC lines or multiple NPC differentiation batches, we have included a NPC purification step in our standard operating protocol in an attempt to further standardize the NPC population and reduce variability between batches and cell lines.

Figure 1: Immunofluorescent labeling of unsorted hPSC-derived NPCs differentiated to the astrocyte lineage. NPCs were exposed to astrocyte medium containing BMP4 (10 ng/ml) and LIF (10 ng/ml) for 28 days, resulting in cells stain positive for the astrocyte-lineage marker GFAP (green) and mostly negative for the early neuronal marker TUJ1 (red).

3. A major rigor issue with this study is the lack of a control group for one of their major conclusions. In the abstract and elsewhere, the authors state that "a combination of LIF and BMP4 directs NPCs to a highly pure population of astrocytes in 28 days". However, there are no control groups to verify whether or not LIF and/or BMP4 are necessary. Many protocols generate hPSC-astrocytes without LIF and BMP4. The experiment should be repeated with and without LIF and BMP4 to confirm whether they are necessary. Are both needed or only one? This is important as there are many divergent protocols to generate astrocytes from hPSCs, and it may explain functional differences between different laboratories.

We fully agree and would like to thank the Reviewer for raising this concern. In order to address this, we have now included a direct comparison of hPSC-derived astrocytes differentiated in astrocyte medium without BMP4 or LIF, with only BMP4, with only LIF, or with both BMP4 and LIF. We found that while a small population of GFAP+ S100B+ double-positive cells emerges when using BMP4 alone, LIF alone or neither, the combination of both BMP4 and LIF greatly enhances the differentiation efficiency. We have added this data to Figure 1-2 and comment on it in the results section (page 17).

4. Many hPSC-astrocyte protocols describe the resultant cells as immature, radial-glia like, even though they express markers such as GFAP/CD44, and maturity changes over time. It is known that immature vs mature astrocytes can have differences in their influence on neurons. Because many of the markers in the RNAseq (Fig. 1) are also expressed in radial glia, it would be important to compare this gene set to biomarkers that have been restricted to immature or mature astrocytes based on previous studies, such as those that performed temporal analysis or single cell analysis of hPSC-astrocytes. This is important as the maturity level of astrocytes may affect results between different studies/labs.

We agree with the Reviewer that cell-type identification on a limited set of markers is indeed problematic. To address this issue, we have now performed single-cell RNA sequencing (scRNA seq) in three independent conditions: a) iPSC-derived astrocyte mono-culture, b) iPSC-derived neuronal mono-culture, and c) co-culture of iPSC-derived astrocytes and neurons. We cross-referenced the full expression profiles of our iPSC-derived neurons and astrocytes to a previously publish human fetal scRNA seq dataset in an attempt to assign cell-type labels (Materials and Methods: 'Culture dissociation and single-cell RNA sequencing' and 'Single-cell RNA sequencing data analysis', page 9-11). Furthermore, these data allowed us to gain further insight into the developmental trajectory of iPSC-derived astrocyte lineage cells when grown in a co-culture with neurons. Data on cell-type identity labels, developmental trajectory and differential expression analysis has been added to the Results section ('Single-cell RNA sequencing confirms astroglial identity and reveals further specification of astrocytes in neuronal co-culture', page 18-21, Figure 2 and Figure 2-1) with corresponding paragraphs in the Discussion section (page 29-30).

5. Regarding the engraftment studies, it has previously been reported in prior publications that hPSC-astrocytes display larger morphology than endogenous rodent astrocytes after engraftment. These prior studies are not cited and should be added. Further, it is not clear in figure 2-1F if the postmortem human astrocyte data was obtained from equal numbers counted among the different samples, or all from one of the samples, etc. as this detailed information is lacking.

We appreciate the opportunity to clarify this important point, and fully agree that prior studies have demonstrated larger morphology of engrafted hPSC-astrocytes compared to endogenous rodent astrocytes. Accordingly, we have now cited prior publications of this finding (Preman et al., 2021; Voronkov et al., 2022; Baranes et al., 2023) (page 22). We used postmortem tissue from three donors (age 61 (n=9), 79 (n=8) and 81 (n=11)) as described in the Materials and Methods sections 'Experimental Model and Subject Details' (page 6) and 'Astrocyte Size Quantification' (page 13). We have now also included related information to the legend of Figure 3-2F.

6. Also, regarding the engraftment studies and in vitro studies, the relevance of measuring human vs rat astrocyte size in vitro and in vivo to test their main hypothesis is not stated. What is the relevance of size to the astrocyte effect on neuron activity? What is the reason to conduct the engraftment studies? This is not explained.

We set out to benchmark our hPSC-derived astrocytes to primary murine astrocytes. Human PSC-derived astrocytes are often used in a co-culture with hPSC-derived neurons in an attempt to further develop these cells and achieve mature electrophysiological properties. Therefore, our main focus was indeed on the development of neuronal electrophysiological properties in a co-culture with rat or hPSC-derived astrocytes. As it has been thoroughly documented that human astrocytes are larger and more complex compared to their murine counterparts, and we observed a corresponding increase in astrocyte size in vitro, we performed xenotransplantations to investigate whether we could recapitulate this effect in an in vivo environment. As this was not the main focus of our studies, we presented this experiment as an Extended Figure (Figure 3-2).

7. Because human astrocytes are larger in vitro, then this may mean that they will have more membrane and ion channels in coculture with neurons if the human and rodent astrocytes are plated at the same cell density, by cell count. Thus, it is likely that the favorable effect observed on neuronal activity is due to the presence of more total cell content, and not due to species differences. The authors need to address this, for example, by testing whether or not the rat vs human astrocytes have different total number of ion channels, or secrete different amount of synaptogenic proteins, for example. Does size matter, and can this be tested? Reviewer 1 raises an interesting hypothesis. We were able to confirm that there is indeed more GFAP surface area in neuron-astrocyte co-cultures with hPSC-derived astrocytes compared to primary rat astrocytes when seeding an identical number of astrocytes to the co-cultures. This data has now been added as Figure 3-1 to the manuscript. It would be interesting to verify if hPSC-derived astrocytes also express a different amount of ion channels or synaptogenic proteins compared to murine astrocytes as suggested by the Reviewer, however, we believe this to be beyond the scope of this study. We have further commented on this in the Results (page 22) and Discussion (page 31) sections.

8. In intro, authors state that NGN2-based neurons require coculture with astrocytes, and they reference Lischka 2018. However, in that paper, they do not use NGN2-neurons and they conclude that mouse (but not human) astrocytes support neuronal function. That finding is in direct contrast with this study and potential reasons for this discrepancy should be discussed or tested.

We appreciate the opportunity to address this discrepancy, and have now rephrased this section along with more extensive citations. Our intent in citing Lischka 2018 was to demonstrate that it has been challenging to obtain functional human astrocytes that are able to support neuronal maturation and functioning at a similar level to murine astrocytes. Therefore, we have now modified this section in an attempt to better explain our reasoning and experimental approach (page 4).

9. In figure 3A, arrows appear to point to synapse on either a map2-negative fiber, or a dim map2-positive fiber underneath the main one. It is not clear how synapses were included/excluded for quantification.

We would like to thank the Reviewer raising this issue. Synapse quantification was performed after thresholding images on the MAP2 signal and counting the co-localization of PSD95, synapsin and MAP2. We have clarified this in the methods section (page 16).

10. Figure 2-1 legends are mislabeled.

We appreciate the Reviewer pointing this out, which has now been corrected.

11. Inconsistency in use of hPSC, hIPSC, hPCS, etc.

We have used both human embryonic stem cell (hESC) and human induced pluripotent stem cell (hIPSC) lines in this manuscript. We use the term hPSC when referring to both hESCs and hiPSCs, while we use hiPSC when referring specifically to induced pluripotent stem cell lines, and hESC when we refer to the embryonic stem cell line.

12. The use of the term "favourably" in the title and elsewhere is confusing. It is recommended that different, more specific word should be used.

This study compares the functionality of primary rat astrocytes to hPSC-derived astrocytes in a co-culture with human neurons. We find that hPSC-derived astrocyte-neuron co-cultures give rise to increased electrophysiological neuronal activity and maturity, combined with the fully human composition of these cultures we feel that 'favourably' is the correct term when using it to summarize the overarching findings of the study. In the main text we have used more specific wording to avoid confusion and describe specific results.

Reviewer 2 Here, the authors derive astrocytes from human iPSCs and compare them to rodent astrocytes for cell culture. There are several elements of this study that show impressive rigor: 1) the use of multiple donor stem cell lines; 2) having a key result replicated in an independent lab; 3) going from mono-culture to co-culture to transplantation into the mouse brain, and taking the latter out an impressive 8 months.

We appreciate the Reviewer's endorsement of our study.

My chief concern is how similar these cells are to actual astrocytes. To that end, I have several questions:

We agree with the Reviewer's concern. Therefore, we have now performed single-cell RNA sequencing (scRNA seq) in three independent conditions: a) iPSC-derived astrocyte mono-culture, b) iPSC-derived neuronal mono-culture, and c) co-culture of iPSC-derived astrocytes and neurons to better characterize the individual cell types. We cross-referenced the full expression profiles of our iPSC-derived neurons and astrocytes to a previously publish human fetal scRNA seq dataset in an attempt to assign cell-type labels (Materials and Methods: 'Culture dissociation and single-cell RNA sequencing' and 'Single-cell RNA sequencing data analysis', page 9-11). Data on cell-type identity labels, developmental trajectory and differential expression analysis has been added to the Results section ('Single-cell RNA sequencing confirms astroglial identity and reveals further specification of astrocytes in neuronal co-culture', page 18-21, Figure 2 and Figure 2-1) with corresponding paragraphs in the Discussion section (page 29-30). We observed a mixed population of glia from the astrocyte lineage in our cultures. In accordance with the scRNAseq results, we now refer to mono-culture astrocytes as 'astroglia', and when grown in the presence of neurons we refer to these cells as 'astrocytes'.

-From Extended Figure 1-1: It would be helpful to see immunofluorescent labeling of astrocyte cultures with NPC markers - how many residual pluripotent cells are found in these cultures? We find no evidence of residual pluripotent stem cells in these cultures, neither by immunofluorescent labeling nor by scRNAseq. This is likely because our starting cell type is a homogenous population of forebrain-patterned neural precursor cells (NPCs), we characterized our NPCs using the markers SOX2 and/or Nestin to confirm neural precursor fate. In correspondence with previously published literature and our scRNAseq data we still observe some SOX2 expression when we perform immunofluorescent labelling on iPSC-derived astroglia, please see Figure 2 below. While we don't consider these to be pluripotent cells, these cells do actively proliferate. The SOX2 labelling in these cells is considerably lower compared to the parental NPCs.

Figure 2: Immunofluorescent labeling of iPSC-derived astrocytes. 28 days after astrocyte differentiation we are able to detect some residual SOX2 signal (red) in GFAP-positive cells (green), consistent with our scRNA seq data.

-In Figure 1F: Given the argument that these cells are similar to cells obtained through other methodology, it would be helpful to see expression of the genes that are canonically used to sort astrocytes, like Hepacam. It would also be very helpful to see additional markers of the NPC/pluripotent state. Is there a way within eNeuro's guidelines to include the complete gene list, or at least anything significantly different? It is very difficult to really assess these cells from the limited information available here. This is exacerbated by the minimal information given on the RNAseq analysis methodology.

We fully agree with the Reviewer that it would be of considerable value to the scientific community to gain insight into the transcriptional profile of hPSC-derived astrocytes established using different methodological approaches. For the cells used in this manuscript we have been able to host the scRNA seq data on an online browser, accessed here: https://cells-test.gi.ucsc.edu/?ds=ipsc-astrocyte-neuron. Upon publication, this dataset will become publicly available. Additionally, we would like to provide an excel file (Table 5) with all differentially expressed genes when comparing mono-culture to co-culture astrocytes. We believe that these data adequately address these concerns and will allow other scientist in the community to compare cell type identify across methodologies.

-Figure 1F: There appears to be a fair amount of difference between the different lines. It would be helpful to see a principle component analysis of the these NPC vs astrocyte lines to get a broader idea of the similarities/differences between lines and differentiation states.

We would like to thank the Reviewer for raising this concern, we have now added a principal component analysis of the bulk RNA sequencing data to Figure 1-3.

Other points:

While it is clear that multiple lines were used in figure 1, it is much less clear if multiple lines were used for all subsequent experiments. Clarification on which line(s) was used for all functional experiments would be very useful; was a particular line chosen, and if so, based on what characteristics? We appreciate the opportunity to address this issue. Cell lines used and total n for each line is specified in each Figure legend. All 4 lines (iPS1 n = 24, iPS2 n = 8, iPS3 n = 6, ES n = 6) were used for the MEA experiments. Whole cell electrophysiological recordings were performed using iPS1 due to the labour-intensive nature of these measurements. Astrocyte analysis in vivo and in vitro was determined using iPS1 and iPS3. Single-cell RNA sequencing experiments were performed using iPS1 to establish the astrocyte, neuron and co-culture samples due to the significant costs associated with the sample preparation and sequencing.

Minor points:

-The authors repeatedly reference "unique hominid features" of their iPSC-derived astrocytes, by which they seem to just mean diameter, as defined by GFAP staining. GFAP staining is not at all a full measure of astrocyte size/diameter, and a change in diameter is not the only feature identified by Oberheim et al. These claims should be specified and toned down. They also repeatedly discuss the complexity of these cells but do not quantify that; complexity should be quantified with something like a Sholl analysis or these claims should be removed (and should be specified as referring to GFAP morphological complexity, as the full morphology of these cells is not being investigated here).

We agree with the Reviewer's comment, and have now modified the text to better reflect that we have only analysed cellular diameter and not morphological complexity (page 22,23).

-Line 458 - the authors state that after 8 months, cells could "still be found in the hippocampus and cortex". This seems to imply that the cells could not be found in other areas, but it is unclear. Was that the only place these cells were found, or the only places the authors looked? We would like to thank the reviewer for pointing out the suggestive nature of our phrasing. We consistently found the xenotransplanted cells in the midbrain, olfactory bulb and cortex, we never found human cells in other brain regions. We have modified the manuscript text to clarify this (page 22).

-Fig 2-1C references "astrocytic domains", which has a specific meaning in regards to astrocytic tiling that does not appear to have been examined here. This should be re-phrased. The labels in this caption are also incorrect.

We agree with the Reviewer and have now modified the labels in this Figure (now Figure 3-2) and changed to wording to better clarify this issue (page 22).

-One key issue in the implementation of these methods as opposed to using rodent cultures is practicality: the ease of generating these cells vs rodent cultures. It would be nice to see the discussion section include information as to time, feasibility, specialized equipment, etc.

Excellent suggestion. We have now included a section in the Discussion to address the issue of practicality and ease of implementation (page 28).

References Baranes K, Hastings N, Rahman S, Poulin N, Tavares JM, Kuan W-L, Syed N, Meik Kunz |, Blighe K, Grant Belgard | T, Kotter MRN (2023) Transcription factor combinations that define human astrocyte identity encode significant variation of maturity and function.

Bullmann T, Kaas T, Ritzau-Jost A, Wöhner A, Kirmann T, Rizalar FS, Holzer M, Nerlich J, Puchkov D, Geis C, Eilers J, Kittel RJ, Arendt T, Haucke V, Hallermann S (2024) Human iPSC-derived neurons with reliable synapses and large presynaptic action potentials. The Journal of Neuroscience 44:e0971232024.

Caiazzo M, Giannelli S, Valente P, Lignani G, Carissimo A, Sessa A, Colasante G, Bartolomeo R, Massimino L, Ferroni S, Settembre C, Benfenati F, Broccoli V (2015) Direct conversion of fibroblasts into functional astrocytes by defined transcription factors. Stem Cell Reports 4:25-36. di Domenico A, Carola G, Calatayud C, Pons-Espinal M, Muñoz JP, Richaud-Patin Y, Fernandez-Carasa I, Gut M, Faella A, Parameswaran J, Soriano J, Ferrer I, Tolosa E, Zorzano A, Cuervo AM, Raya A, Consiglio A (2019) Patient-Specific iPSC-Derived Astrocytes Contribute to Non-Cell-Autonomous Neurodegeneration in Parkinson's Disease. Stem Cell Reports 12:213-229 Available at: http://www.ncbi.nlm.nih.gov/pubmed/30639209 [Accessed January 20, 2019].

Hulme AJ, Maksour S, St-Clair Glover M, Miellet S, Dottori M (2022) Making neurons, made easy: The use of Neurogenin-2 in neuronal differentiation. Stem Cell Reports 17:14-34.

Jovanovic VM et al. (2023) A defined roadmap of radial glia and astrocyte differentiation from human pluripotent stem cells. Stem Cell Reports 18:1701-1720 Available at: http://www.ncbi.nlm.nih.gov/pubmed/37451260 [Accessed July 23, 2023].

Kondo T et al. (2016) Modeling Alexander disease with patient iPSCs reveals cellular and molecular pathology of astrocytes. Acta Neuropathol Commun 4:69 Available at: http://actaneurocomms.biomedcentral.com/articles/10.1186/s40478-016-0337-0 [Accessed July 14, 2016].

Krencik R, Seo K, van Asperen J V., Basu N, Cvetkovic C, Barlas S, Chen R, Ludwig C, Wang C, Ward ME, Gan L, Horner PJ, Rowitch DH, Ullian EM (2017) Systematic Three-Dimensional Coculture Rapidly Recapitulates Interactions between Human Neurons and Astrocytes. Stem Cell Reports 9:1745-1753 Available at: http://www.cell.com/article/S2213671117304812/fulltext [Accessed October 17, 2022].

Preman P, TCW J, Calafate S, Snellinx A, Alfonso-Triguero M, Corthout N, Munck S, Thal DR, Goate AM, De Strooper B, Arranz AM (2021) Human iPSC-derived astrocytes transplanted into the mouse brain undergo morphological changes in response to amyloid-β plaques. Mol Neurodegener 16:68.

Sloan SA, Darmanis S, Huber N, Khan TA, Birey F, Caneda C, Reimer R, Quake SR, Barres BA, Paşca SP (2017) Human Astrocyte Maturation Captured in 3D Cerebral Cortical Spheroids Derived from Pluripotent Stem Cells. Neuron 95:779-790.e6 Available at: https://linkinghub.elsevier.com/retrieve/pii/S0896627317306839.

Tcw J, Wang M, Pimenova AA, Bowles KR, Hartley BJ, Lacin E, Machlovi SI, Abdelaal R, Karch CM, Phatnani H, Slesinger PA, Zhang B, Goate AM, Brennand KJ (2017) An Efficient Platform for Astrocyte Differentiation from Human Induced Pluripotent Stem Cells. Stem Cell Reports 9:600-614 Available at: http://www.ncbi.nlm.nih.gov/pubmed/28757165 [Accessed August 7, 2017].

Voronkov DN, Stavrovskaya A V., Guschina AS, Olshansky AS, Lebedeva OS, Eremeev A V., Lagarkova MA (2022) Morphological Characterization of Astrocytes in a Xenograft of Human iPSC-Derived Neural Precursor Cells. Acta Naturae 14:100-108.

Wang S, Rhijn J-R van, Akkouh I, Kogo N, Maas N, Bleeck A, Ortiz IS, Lewerissa E, Wu KM, Schoenmaker C, Djurovic S, van Bokhoven H, Kleefstra T, Nadif Kasri N, Schubert D (2022) Loss-of-function variants in the schizophrenia risk gene SETD1A alter neuronal network activity in human neurons through the cAMP/PKA pathway. Cell Rep 39:110790.
