# Supplementary figures and images for: Human Pluripotent Stem Cell-Derived Astrocyte Functionality Compares Favorably with Primary Rat Astrocytes
Source: eNeuro. 2024 Sep 12;11(9):ENEURO.0148-24.2024. doi: 10.1523/ENEURO.0148-24.2024 (PMC11404293; doi:10.1523/ENEURO.0148-24.2024)

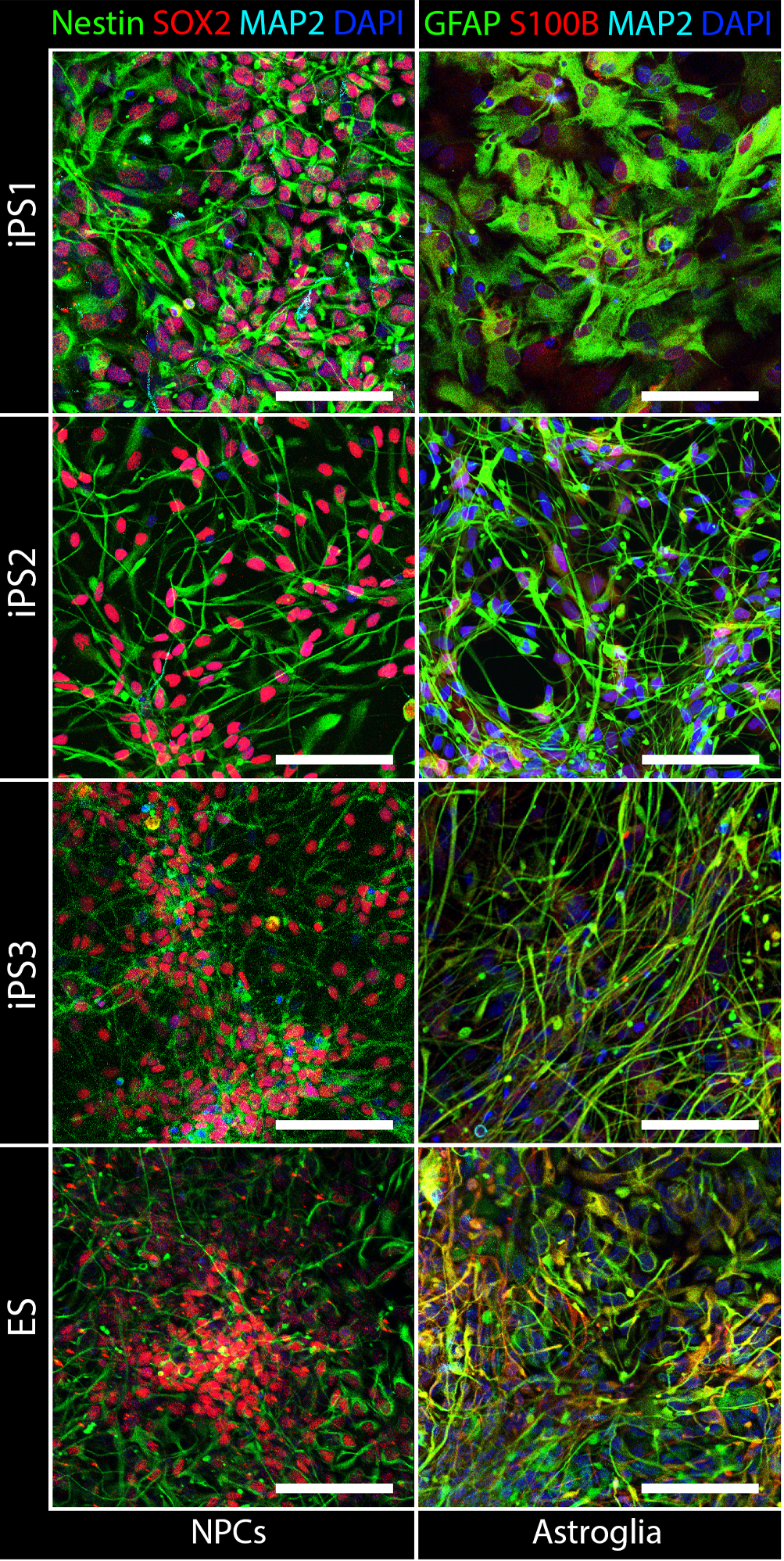

Supplement: Figure 1-1 — Immunofluorescent labeling of NPCs and their derived astroglia cultures. NPCs stain positive for Nestin (green) and SOX2 (red) and negative for MAP2 (cyan). Astroglia stain positive for GFAP (green) and S100B (red) and negative for MAP2 (cyan) (scale bar = 50 μm). Download Figure 1-1, TIF file. [file eneuro-11-ENEURO.0148-24.2024-s001.tif]

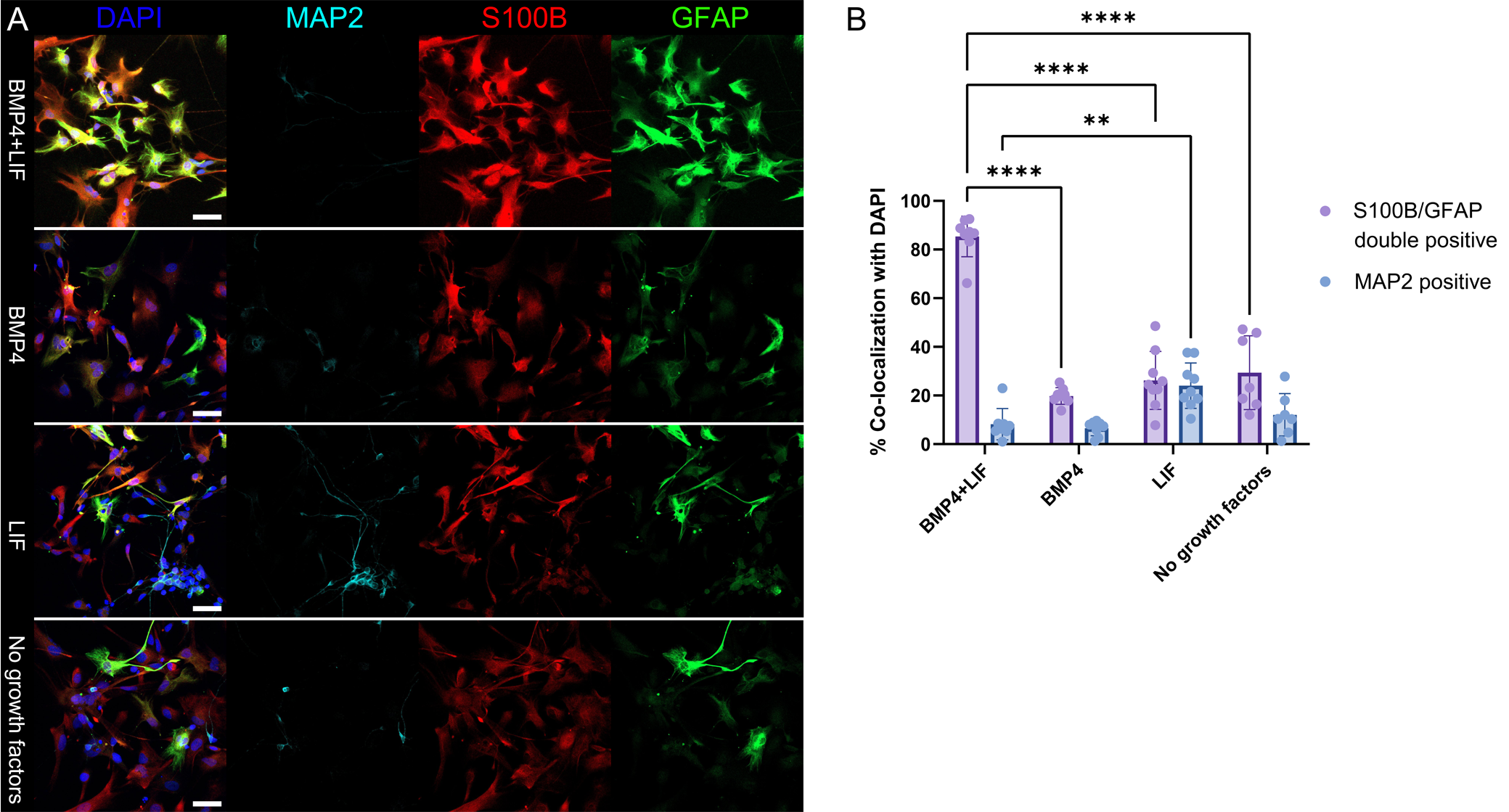

Supplement: Figure 1-2 — BMP4 and LIF are required for efficient astroglia differentiation. (A) Representative images of hPSC-derived NPCs (iPS1) exposed to astrocyte medium containing BMP4 (10 ng/ml) and LIF (10 ng/ml), only BMP4 (10 ng/ml), only LIF (10 ng/ml) or no additional growth factors during a 4-week period (scale bar = 50 µm). Cells were stained with GFAP, S100B and MAP2 to confirm astroglial identify and evaluate neuronal contamination. (B) Quantification of cells double positive for S100B and GFAP (astroglia) and MAP2 (neuronal cells). Medium containing both BMP4 and LIF is more efficient (2-way ANOVA, P<0.001) in differentiating NPCs towards an astroglial fate (85.39% ± 2.94 (BMP4 and LIF), 19.80% ± 1.20 (BMP4), 26.18 % ± 3.97 (LIF), 29.40 % ± 5.73 (no growth factors)), medium containing only LIF gave rise to more neuronal cells (2-way ANOVA, P<0.01) (8.19% ± 2.27 (BMP4 and LIF), 6.50% ± 1.07 (BMP4), 24.08 % ± 3.11 (LIF), 12.07 % ± 3.30 (no growth factors)). Download Figure 1-2, TIF file. [file eneuro-11-ENEURO.0148-24.2024-s002.tif]

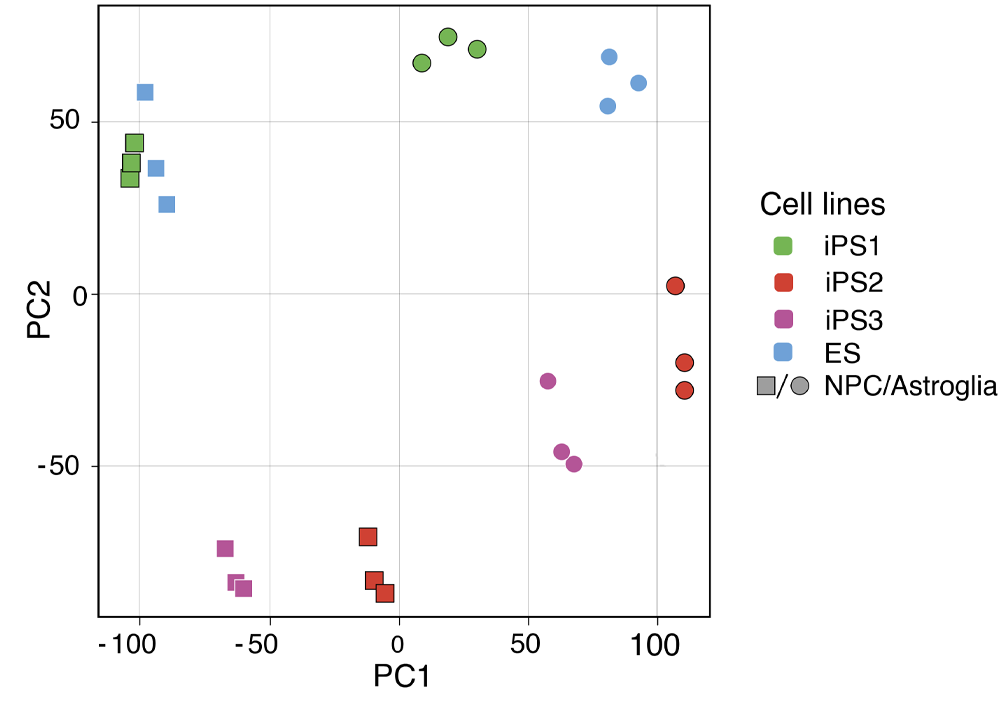

Supplement: Figure 1-3 — Principal component analysis of bulk RNA sequencing results. PCA plot displaying all samples used for bulk RNA sequencing. Astroglia samples are depicted as circles, NPC samples as squares. Cell lines are depicted in different colors: iPS1 (purple), iPS2 (green), iPS3 (red) and ES (blue). Download Figure 1-3, TIF file. [file eneuro-11-ENEURO.0148-24.2024-s003.tif]

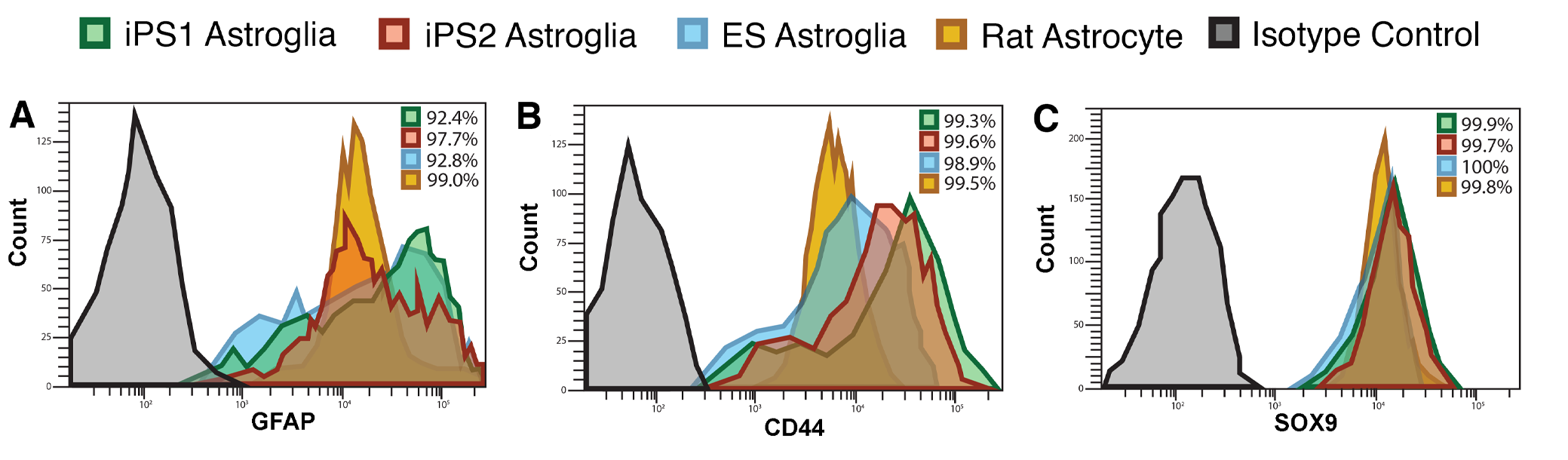

Supplement: Figure 1-4 — Flow-cytometry quantification of astrocyte markers. Fluorescence intensity histogram plots for iPS 1-, iPS 2- and embryonic stem cell (ES)-derived astroglia compared to primary rat astrocytes for GFAP (A), CD44 (B) and SOX9 (C). Download Figure 1-4, TIF file. [file eneuro-11-ENEURO.0148-24.2024-s004.tif]

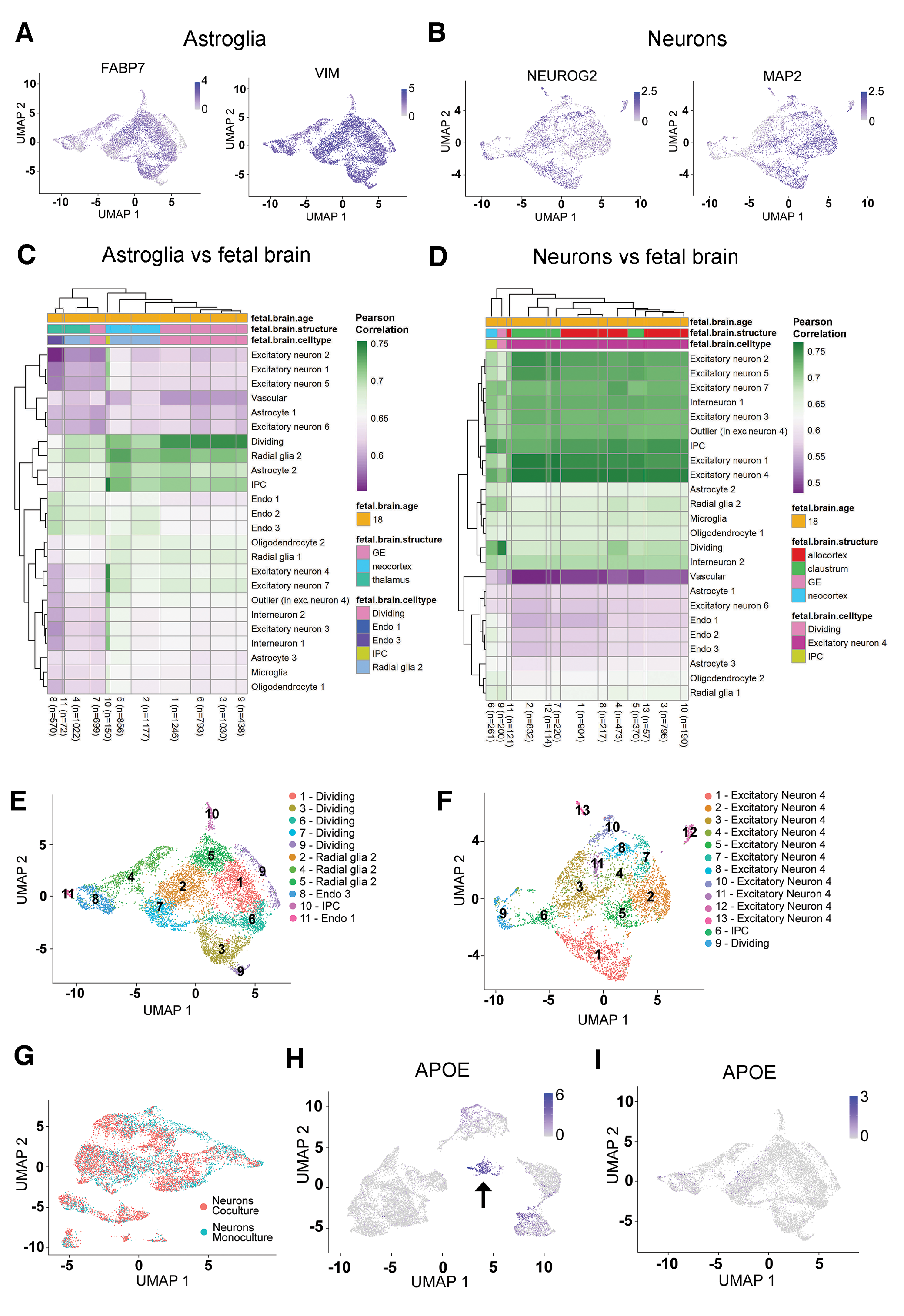

Supplement: Figure 2-1 — Single-cell RNA sequencing data from astroglia and neuron mono-culture samples. (A) Astroglia sample (iPS1) shows homogenous expression of known astrocyte markers, e.g. FABP7 and VIM. (B) Ngn2-neuron sample (iPS1) shows homogenous expression of known neuronal markers, e.g. MAP2 and NEUROG2. (C) Heatmap showing Pearson’s correlation between scRNA seq cell-clusters of the astroglia sample and primary fetal brain tissue. (D) Heatmap showing Pearson’s correlation between scRNA seq cell-clusters of the Ngn2-neuron sample and primary fetal brain tissue. (E) UMAP projection of the astroglia sample with transferred cell type labels with the highest correlation from primary human brain tissue. (F) UMAP projection of the Ngn2-neuron sample with transferred cell type labels with the highest correlation from primary brain tissue. (G) UMAP projection of integrated Ngn2-neuron sample with original sample identity indicated in green (monoculture) or red (coculture). (H, I) APOE expression is upregulated in astroglia under coculture conditions and mostly expressed in a single cluster (cluster 10, “Radial glia”, arrow) (H), in a culture with only astrocytes (I) APOE expression is lower. Download Figure 2-1, TIF file. [file eneuro-11-ENEURO.0148-24.2024-s005.tif]

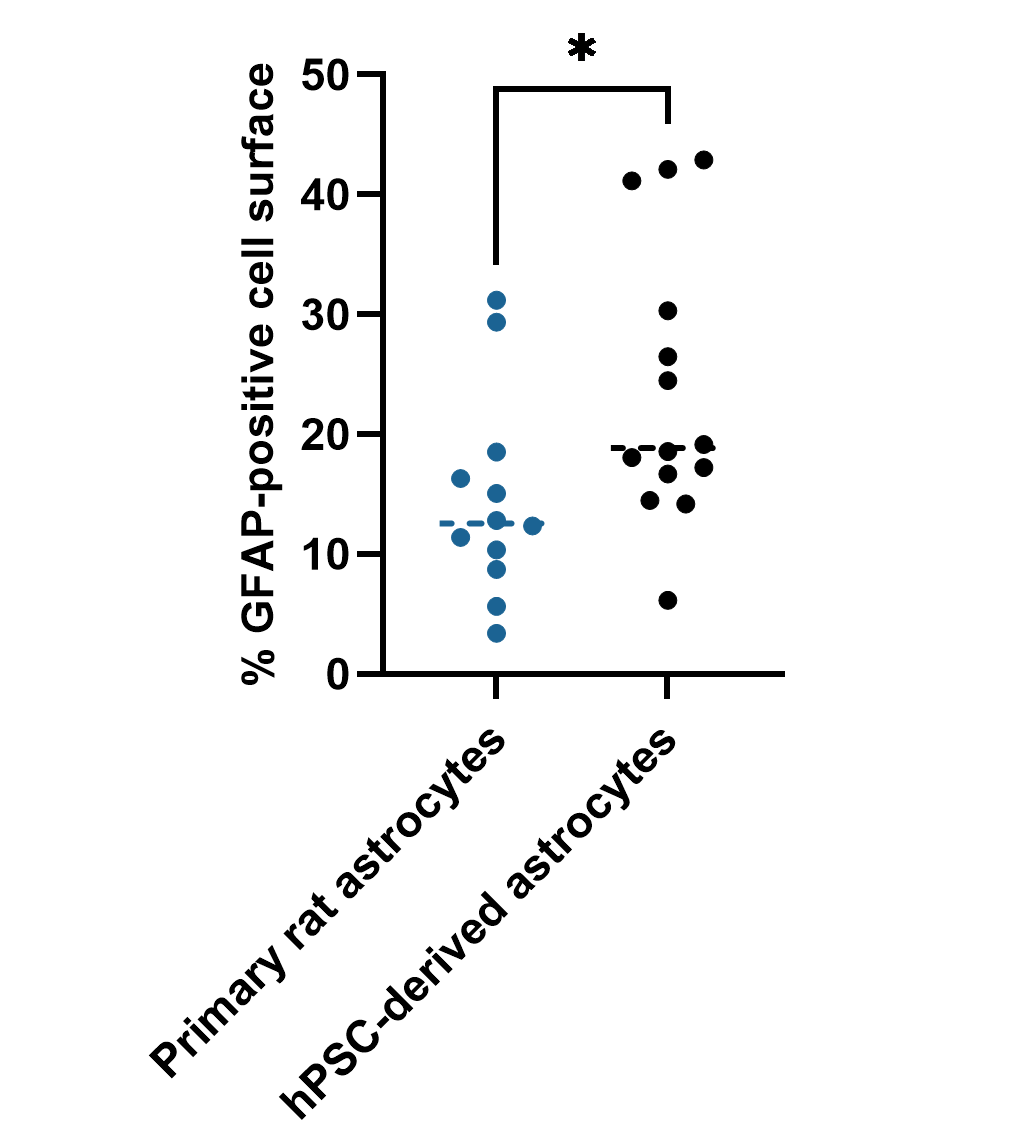

Supplement: Figure 3-1 — Percentage of GFAP-positive cell surface of the total area in astrocyte-neuron cocultures. The surface area percentage of primary rat astrocytes (n=12) and hPSC-derived astrocytes (iPS1, n=14). Download Figure 3-1, TIF file. [file eneuro-11-ENEURO.0148-24.2024-s006.tif]

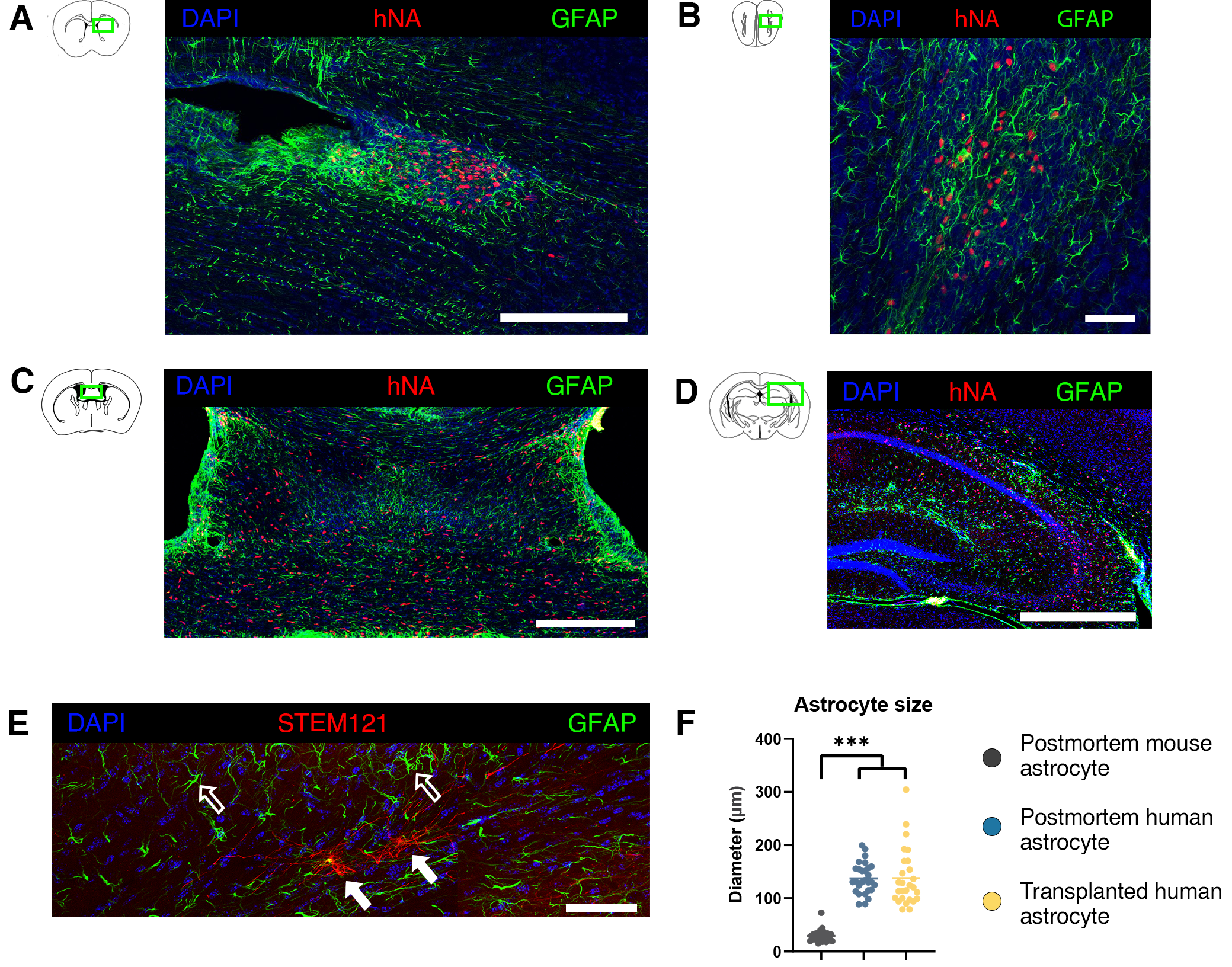

Supplement: Figure 3-2 — Human PSC-derived astrocytes integrate in the mouse brain after neonatal xenotransplantation. (A) 4 weeks after xenotransplantation hPSC-derived astrocytes (iPS3, human nuclear antigen (hNA), red) are mainly found in the subventricular zone of the lateral ventricles (scale bar = 200 μm). (B) Human PSC-derived astrocytes (iPS3, hNA, red) in the olfactory bulb of a 4-week-old mouse (scale bar = 50 μm). (C) Human PSC-derived astrocytes (iPS3, hNA, red) self-organize into astrocytic domains 8 weeks after xenotransplantation (scale bar = 300 μm). (D) Human PSC-derived astrocytes (iPS3, hNA, red) populate the mouse hippocampus 8 months after xenotransplantation (scale bar = 500 μm). (E) Human PSC-derived astrocytes (red, solid arrows) are larger and more complex compared to their rodent counterpart (green, open arrows) in an identical in vivo environment (scale bar = 50 μm). (F) Cell size quantification (maximum diameter) of postmortem mouse astrocytes (black, n = 26), postmortem human astrocytes (blue, n = 28 (3 donors, age: 61 (n=9), 79 (n=8) and 81 (n=11)) and xenotransplanted hPSC-derived astrocytes (iPS3, yellow, n = 27). Download Figure 3-2, TIF file. [file eneuro-11-ENEURO.0148-24.2024-s007.tif]

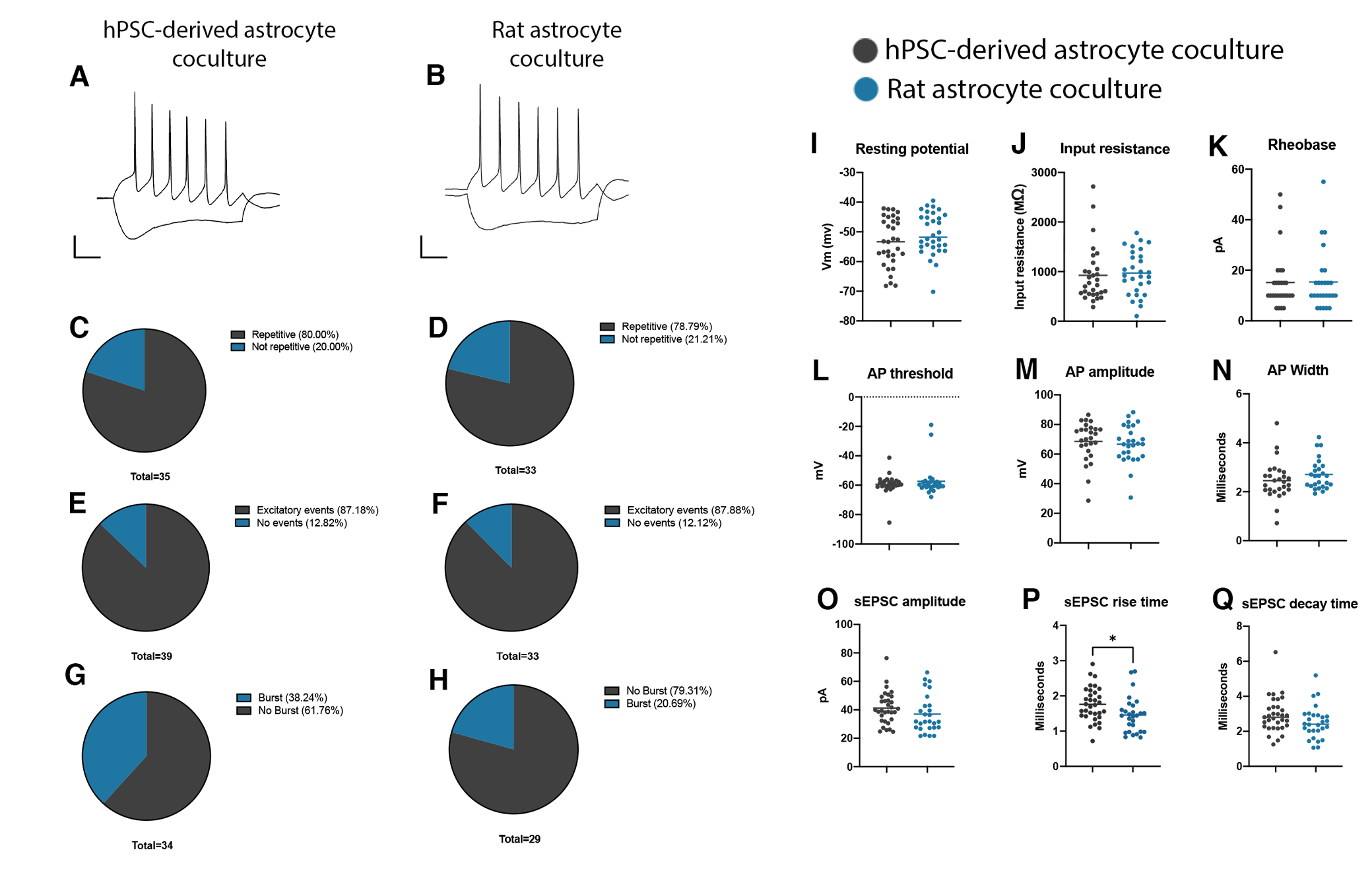

Supplement: Figure 4-1 — Whole-cell electrophysiological recordings of two-week old neuronal cocultures with hPSC-derived or rodent astrocytes. (A, B) Representative traces of evoked action potentials in cultures with hPSC-derived (A) or rat (B) astrocytes. (C, D) Percentage of neurons able to fire repetitive action potentials upon current injection in cultures with hPSC-derived (C) or rat (D) astrocytes. (E, F) Percentage of neurons that receive spontaneous synaptic input in cultures with hPSC-derived (E) or rat (F) astrocytes. (G, H) Percentage of neurons that received bursts of post synaptic currents in cultures with hPSC-derived (G) or rat (H) astrocytes, this percentage was non-significantly increased in cultures with hPSC-derived astrocytes. (I – N) Resting membrane potential (I), input resistance (J), rheobase (K), AP threshold (L), AP amplitude (M) and AP width (N) were similar in cocultures with hPSC-derived (black) or rat (blue) astrocytes (n= 29 (hPSC, iPS1) and 30 (rat) cells). (O) sEPSC amplitude was similar in both conditions. (P) sEPSC rise time was slower in cocultures with hPSC-derived astrocytes (two-tailed t-test, P<0.05). (Q) No differences were found in the decay time of sEPSC (n= 27 (hPSC, iPS1) and 32 (rat) cells). Download Figure 4-1, TIF file. [file eneuro-11-ENEURO.0148-24.2024-s008.tif]

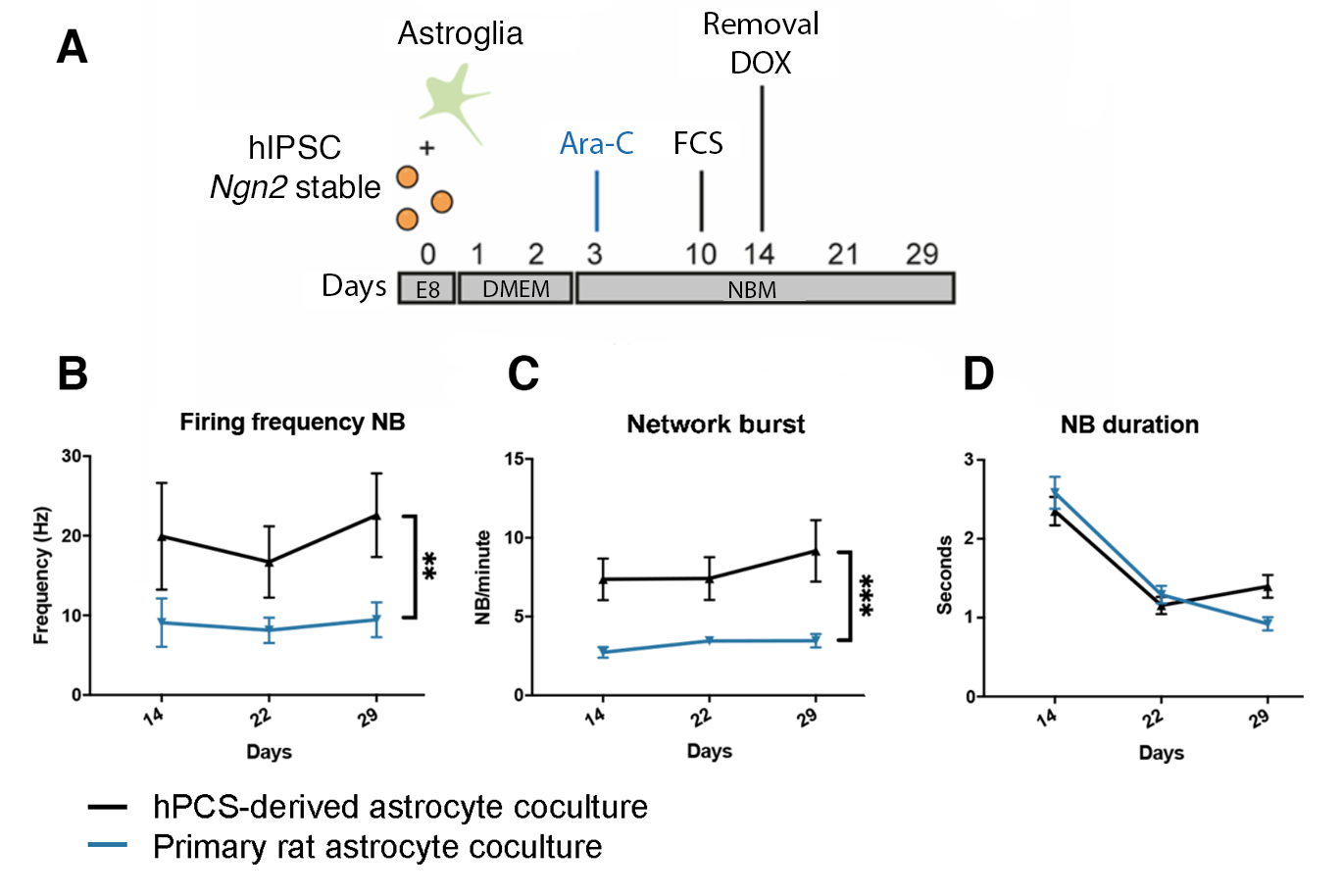

Supplement: Figure 5-1 — Implementation of hPSC-derived astrocytes in an independent laboratory. (A) Experimental setup of neuronal coculture. Human iPSCs are plated together with astrocytes in a coculture and Ngn2 overexpression is induced in iPSCs using doxycycline to initiate neuronal differentiation. (B - D) Analysis of Ngn2-neuronal cocultured with hPSC-derived astrocytes (iPS1, n = 10) or primary rat astrocytes (n = 9). Mean firing frequency within NBs (B) and NB rate per minute (C) is increased in hPSC-derived astrocyte cocultures, while network burst duration is similar across conditions (D). Download Figure 5-1, TIF file. [file eneuro-11-ENEURO.0148-24.2024-s009.tif]
